# Lamin B1 loss promotes lung cancer development and metastasis by epigenetic derepression of RET

**DOI:** 10.1084/jem.20181394

**Published:** 2019-04-23

**Authors:** Yanhan Jia, Joaquim Si-Long Vong, Alina Asafova, Boyan K. Garvalov, Luca Caputo, Julio Cordero, Anshu Singh, Thomas Boettger, Stefan Günther, Ludger Fink, Till Acker, Guillermo Barreto, Werner Seeger, Thomas Braun, Rajkumar Savai, Gergana Dobreva

**Affiliations:** 1 Max Planck Institute for Heart and Lung Research, Member of the German Center for Lung Research, Bad Nauheim, Germany; 2 Anatomy and Developmental Biology, Centre for Biomedicine and Medical Technology Mannheim (CBTM) and European Center for Angioscience (ECAS), Medical Faculty Mannheim, Heidelberg University, Mannheim, Germany; 3 Microvascular Biology and Pathobiology, European Center for Angioscience (ECAS), Medical Faculty Mannheim, Heidelberg University, Mannheim, Germany; 4 Institute of Neuropathology, Justus Liebig University, Giessen, Germany; 5 Institute of Pathology and Cytology, Überregionale Gemeinschaftspraxis für Pathologie (ÜGP), Wetzlar, Germany; 6 Department of Internal Medicine, Justus Liebig University, Member of the German Center for Lung Research (DZL), Giessen, Germany; 7 Medical Faculty, J.W. Goethe University Frankfurt, Frankfurt, Germany

## Abstract

Although abnormal nuclear structure is an important criterion for cancer diagnostics, remarkably little is known about its relationship to tumor development. Here we report that loss of lamin B1, a determinant of nuclear architecture, plays a key role in lung cancer. We found that lamin B1 levels were reduced in lung cancer patients. Lamin B1 silencing in lung epithelial cells promoted epithelial–mesenchymal transition, cell migration, tumor growth, and metastasis. Mechanistically, we show that lamin B1 recruits the polycomb repressive complex 2 (PRC2) to alter the H3K27me3 landscape and repress genes involved in cell migration and signaling. In particular, epigenetic derepression of the RET proto-oncogene by loss of PRC2 recruitment, and activation of the RET/p38 signaling axis, play a crucial role in mediating the malignant phenotype upon lamin B1 disruption. Importantly, loss of a single lamin B1 allele induced spontaneous lung tumor formation and RET activation. Thus, lamin B1 acts as a tumor suppressor in lung cancer, linking aberrant nuclear structure and epigenetic patterning with malignancy.

## Abstract (precis)

Jia et al. demonstrate that lamin B1 acts as a tumor suppressor in lung cancer. Lamin B1 loss promotes lung cancer development and metastasis by loss of PRC2 recruitment to chromatin and activation of the RET/p38 signaling axis.

## Graphical Abstract

**Figure:**
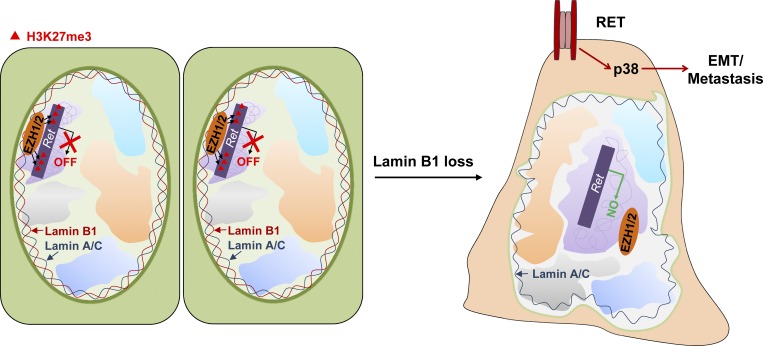

## Introduction

Lung cancer is the leading cause of cancer-related death worldwide (Siegel et al., 2017), mainly due to its high propensity to metastasize rapidly. Lung tumors are divided into two major histopathological groups: small-cell lung cancer (SCLC) and non–small-cell lung cancer (NSCLC). NSCLC, which accounts for ∼80% of all cases, is subdivided into adenocarcinoma, squamous cell carcinoma (SCC), and large-cell carcinoma. A key characteristic and important diagnostic criterion for lung cancer and other neoplasias is alteration of the nuclear structure, including characteristic changes in nuclear shape and size, the number of nucleoli and nuclear bodies, chromatin appearance, and a “polymorphic” nuclear envelope with abnormal nuclear blebs (Zink et al., 2004; Chow et al., 2012). It has been shown that collapse of the nuclear envelope in NSCLC cells triggers extensive DNA damage and can be used as a valuable biomarker for genomic instability in lung tumors (Hatch et al., 2013). The nuclear envelope, which is an important determinant of nuclear structure, shape, and genome integrity, is composed of nuclear membranes, nuclear lamina, and nuclear pore complexes (Bukata et al., 2013; Van Bortle and Corces, 2013). The nuclear lamina is located between the inner nuclear membrane and the peripheral heterochromatin and consists of a proteinaceous meshwork of intermediate filaments, the lamins (Butin-Israeli et al., 2012; Burke and Stewart, 2013). There are two separate classes of lamins, A-type and B-type. While B-type lamins are present throughout development, A-type lamins are expressed only after commitment of cells to a particular differentiation pathway (Stewart and Burke, 1987), suggesting distinct molecular functions of A- and B-type lamins in different cell types. All lamins share a common structure and form coiled-coil dimers that associate in protofilaments and higher-order lamin structures (McKeon et al., 1986; Dittmer and Misteli, 2011). However, high-resolution confocal microscopy demonstrated that the different type of lamins form distinct meshworks, which show low colocalization, further suggesting distinct functions. The major fraction of lamins is found at the nuclear lamina, to support the nuclear envelope and provide anchorage sites for chromatin (Shimi et al., 2008). Genome-wide profiling of lamin B1 binding identified large lamina-associated domains (LADs), consisting of megabase-sized, relatively gene-poor, and repressive chromatin domains, that dynamically associate with the nuclear lamina (Guelen et al., 2008; Reddy et al., 2008; Peric-Hupkes et al., 2010). The majority of genes associated with lamin B1 are transcriptionally inactive and enriched in repressive histone marks such as H3K27me3 and H3K9me2/3 (Reddy et al., 2008; Wen et al., 2009). In contrast, A-type lamins associate with both hetero- and euchromatin (Shimi et al., 2008; Gesson et al., 2016). In addition to their key function in regulating nuclear structure stability (Sullivan et al., 1999; Vergnes et al., 2004; Shimi et al., 2008), chromatin organization and gene positioning (Guelen et al., 2008; Reddy et al., 2008), lamins play a key role in the regulation of DNA replication and repair (Jenkins et al., 1993; Moir et al., 2000; Butin-Israeli et al., 2013), cell cycle progression, and cell proliferation and differentiation (Burke and Stewart, 2013). Consistently, mutations in lamins lead to a broad spectrum of diseases (Schreiber and Kennedy, 2013). Changes in the expression of lamins have been linked to various tumor entities; however, the relationship appears to be complex and tumor-type specific, and direct evidence for their function in cancer is lacking (Butin-Israeli et al., 2012; Burke and Stewart, 2013; Hutchison, 2014).

Global epigenetic reprogramming is another hallmark of cancer cells. Polycomb group (PcG) proteins are epigenetic repressors with a key function in cancer (Dawson and Kouzarides, 2012; Conway et al., 2015; Comet et al., 2016). Two major polycomb repressive complexes (PRCs) have been identified: PRC1 and PRC2. PRC1 ubiquitylates histone H2A on Lys119 (Wang et al., 2004a), whereas PRC2 catalyzes the mono-, di-, and trimethylation of H3 on Lys27 (Cao et al., 2002). Generally, the H3K27me2/3 marks act as a docking site for the chromobox-domain protein subunits of the PRC1 complexes, leading to PRC1 recruitment and polycomb-mediated chromatin compaction (Wang et al., 2004b). This, in turn, reduces the accessibility of chromatin to transcription factors and chromatin remodelers, resulting in transcriptional repression. EED, SUZ12, and one of the two histone H3K27 methyltransferases, EZH1 or EZH2 (Cao et al., 2002; Czermin et al., 2002), are core components of the PRC2 complex. Both gain and loss of PRC2 function, as a result of mutations in these core components, have been linked to cancer initiation, progression, and metastasis, suggesting that the role of the PRC2 complex in tumorigenesis is multifaceted and highly cell type specific (Dawson and Kouzarides, 2012; Conway et al., 2015; Comet et al., 2016). In NSCLC models, PRC2 suppresses epithelial–mesenchymal transition (EMT), but the effects of its modulation are dependent on the genetic context of the tumors (Serresi et al., 2016). The levels of EZH2 and other PRC2 components have been reported to be high in SCLC (Sato et al., 2013), and recently a therapeutic strategy involving chemotherapy combined with EZH2 inhibitors was suggested to prevent chemotherapy resistance of SCLC (Gardner et al., 2017). In stark contrast, loss of EZH2 induces multidrug resistance of acute myeloid leukemia (Göllner et al., 2017). The highly context-dependent role of the PRC2 complex in cancer illustrated by these studies emphasizes the need to consider systemic and long-term treatment with EZH2 inhibitors with caution and calls for a more precise understanding of the downstream mechanisms underlying PRC2 function in cancer.

In our paper, we uncover a key function of lamin B1 as a tumor suppressor in lung cancer. Mechanistically, we show that lamin B1 recruits the PRC2 complex to repress genes involved in cell migration and signaling. We found that derepression of the rearranged during transfection (RET) proto-oncogene and activation of the RET/p38 signaling axis mediates the malignant phenotype upon lamin B1 loss. Thus, lamin B1 plays a key role in lung cancer development and progression, providing a molecular link between altered nuclear morphology, aberrant epigenetic patterning, and the malignant phenotype.

## Results

### Lamin B1 levels are reduced in lung cancer patients and lower lamin B1 expression correlates with higher lung cancer grade

**Figure 1.**
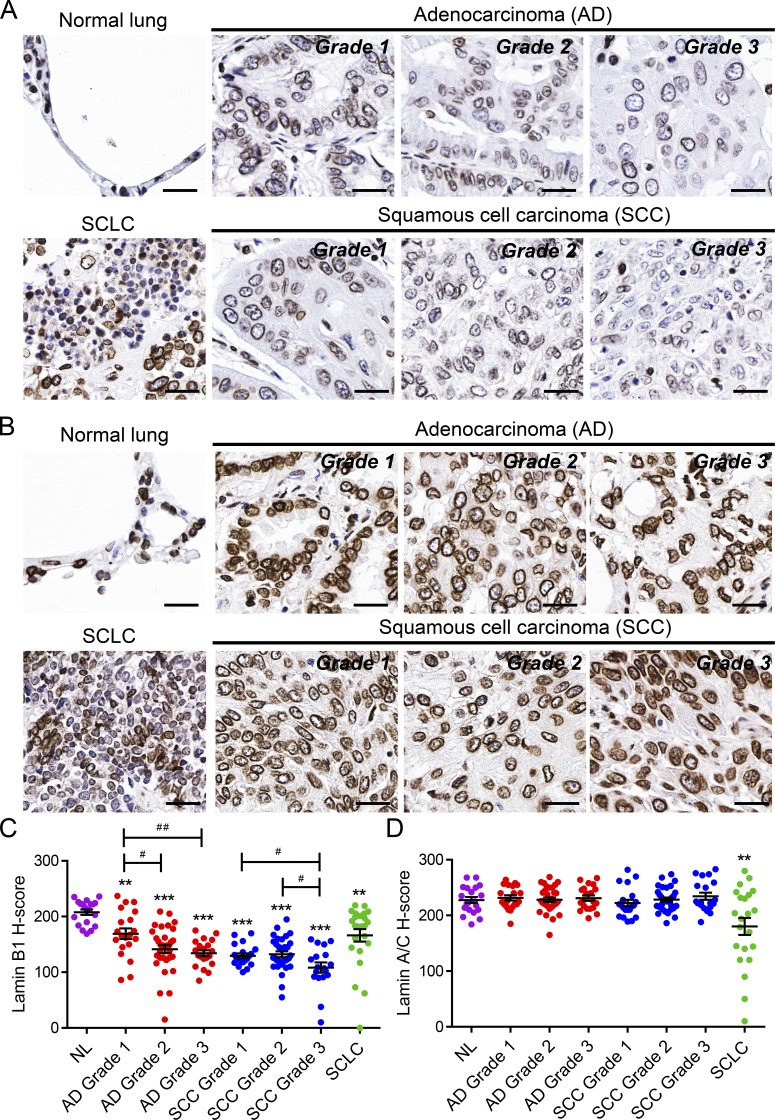
**Reduced lamin B1 levels in lung cancer patients. (A and B)** IHC of representative tissue samples from different types and grades of lung tumors from a tissue microarray stained with an anti–lamin B1 (A) and anti–lamin A/C (B) antibody. Scale bars, 25 µm. **(C and D)** Relative staining intensity (H-score) for lamin B1 (C) and lamin A/C (D) in lung tumors of different types and grades. *n* = 70 for adenocarcinoma ([AD] *n* = 20 grade 1, *n* = 30 grade 2, *n* = 20 grade 3); *n* = 69 for SCC (*n* = 16 grade 1, *n* = 33 grade 2, *n* = 20 grade 3); *n* = 22 for SCLC; and *n* = 20 for normal lung tissue (NL). Statistical analysis was performed using Student’s *t* test with two-tailed distribution. Data are mean ± SEM. **, P < 0.01; ***, P < 0.001 versus normal lung tissue. #, P < 0.05; ##, P < 0.01.

To determine whether irregular expression of lamins may contribute to the abnormalities observed in the nuclei of lung cancer cells and may be linked to lung tumor initiation and progression, we first analyzed a human lung tissue microarray comprising different types and grades of lung cancer specimens (Fig. 1 and Fig. S1). Immunostainings using two different antibodies revealed lower expression of lamin B1 in SCLC and a progressive loss of lamin B1 in high-grade adenocarcinoma and SCC, which have a significantly worse prognosis compared with lower grade tumors (Fig. 1, A and C; and Fig. S1, A and B). Grade I cancer cells expressed higher levels of nuclear lamin B1, both in terms of intensity and percentage of positive cells, whereas in grade II and especially in grade III tumors, much fewer cells expressed lamin B1, typically at substantially lower levels (Fig. 1, A and C). In contrast, no differences were observed in lamin A expression between normal lung tissue and all subtypes and grades of NSCLC, except for a decrease in SCLC (Fig. 1, B and D). In grade II and, in particular, in grade III tumors, we observed clusters of cells highly positive for lamin B1 but negative for lamin A, which stained positive for the immune/inflammatory marker CD45 (Fig. S1, C and D). Thus, lamin B1 levels are significantly reduced in tumor cells of lung cancer patients but are high in immune cells, which have been shown to exhibit stage-dependent accumulation in human lung tumors (Banat et al., 2015) decrease of lamin B1 levels in lung cancer compared with nonmalignant lung cells/tissues was confirmed by Western blot analysis of mouse lung epithelial (MLE12) cells and normal human bronchial epithelium B2B (BEAS-2B) cells, compared with the highly aggressive, metastatic mouse Lewis lung carcinoma (LLC1) cells, as well as H69 human SCLC cells (Fig. S1 E). Interestingly, LLC1 cells showed abnormal nuclear shape and lamin B1 localization (Fig. S1 F). Together, these findings support the notion that loss of lamin B1 may play a role in promoting lung cancer initiation, progression, and malignancy.

### Lamin B1 loss promotes EMT and metastasis

**Figure 2.**
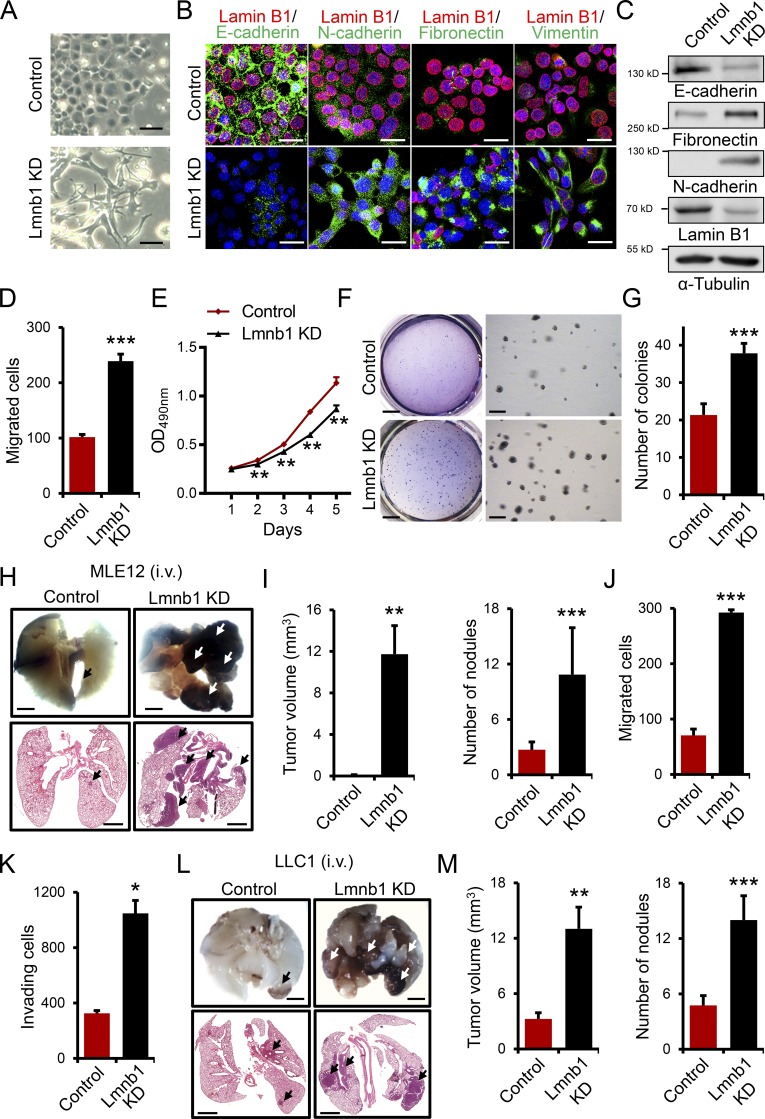
**Lamin B1 silencing promotes EMT, tumor growth, and metastasis. (A)** Images of control and lamin B1 KD (Lmnb1 KD) MLE12 cells showing morphological changes after lamin B1 silencing. Scale bars, 50 µm. **(B and C)** Immunostainings (B) and Western blot analysis (C) for EMT markers in control and lamin B1 KD MLE12 cells. Cell nuclei were labeled by DAPI. Scale bars, 20 µm. **(D)** Boyden chamber migration assay with control and lamin B1 KD MLE12 cells (*n* = 4). **(E)** MTS cell proliferation assay to determine the effect of lamin B1 loss on cell proliferation (*n* = 8). **(F)** Soft agar assay to assess the anchorage independent cell growth of control and lamin B1 KD MLE12 cells. Higher magnifications (right panels) show the increased colony size upon lamin B1 loss (*n* = 6). Scale bars of left panels, 4 mm; scale bars of right panels, 0.5 mm. **(G)** Quantification of the number of colonies per field in the soft agar assay. **(H and I)** Control and lamin B1 KD MLE12 cells were injected intravenously into the tail vein of BALB/c nu/nu mice. After 24 d, mice were sacrificed and examined for tumor metastases in the lungs. Macroscopic appearance (H, top) and H&E (H, bottom) staining of representative lungs. Arrows indicate metastatic nodules. Scale bars, 2 mm. **(I)** Quantification of the microscopic tumor volume and metastatic nodules. *n* = 9 for the control, 8 for the Lmnb1 KD group (MLE12). **(J and K)** Boyden chamber migration (J) and invasion (K) assay with control and lamin B1 KD LLC1 cells (*n* = 4). **(L and M)** Control and lamin B1 KD LLC1 cells were injected intravenously into the tail vein of C57BL/6 mice for 20 d. Macroscopic appearance (L, top) and H&E (L, bottom) staining of representative lungs. Arrows indicate metastatic nodules. Scale bars, 2 mm. **(M)** Quantification of the macroscopic tumor volume and metastatic nodules. *n* = 8 for the control and Lmnb1 KD groups (LLC1). Results (B–G, J, and K) are representative of a minimum of three independent experiments. Statistical analysis was performed using Student’s *t* test with two-tailed distribution. Data are mean ± SEM. *, P < 0.05; ** P < 0.01; ***, P < 0.001.

EMT is a crucial event during tumor progression and metastasis (De Craene and Berx, 2013). To determine whether lamin B1 plays a role in EMT, we depleted lamin B1 in MLE12 cells by lentivirus-driven shRNA-mediated silencing and clustered regularly interspaced short palindromic repeats (CRISPR)/Cas9 gene editing (Fig. S2, A and B). While control MLE12 cells maintained a cobblestone-like, epithelial appearance in culture, lamin B1–depleted MLE12 cells developed a spindle-shaped morphology (Fig. 2 A and Fig. S2 B). We detected significant downregulation of the epithelial marker E-cadherin and upregulation of the mesenchymal markers fibronectin, vimentin, and N-cadherin in lamin B1 knockdown (KD) cells (Fig. 2, B and C). Importantly, lamin B1–depleted MLE12 cells possessed a significantly higher migratory capacity in scratch wound healing and Boyden chamber–based migration assays (Fig. 2 D and Fig. S2 C). The role of lamin B1 in cell proliferation appears to be complex and cell type specific (Butin-Israeli et al., 2012). Thus, we next tested the role of lamin B1 silencing on cell proliferation and anchorage-independent growth. Interestingly, while lamin B1 silencing led to decreased cell proliferation in two-dimensional adherent monolayer cultures (Fig. 2 E), the capacity of MLE12 cells to form colonies in soft agar as a measure of anchorage-independent growth, one of the hallmarks of malignant transformation (Hanahan and Weinberg, 2011), was markedly increased after downregulation of lamin B1 (Fig. 2, F and G). Furthermore, the individual colony size was increased by lamin B1 depletion (Fig. 2 F). To further examine the role of lamin B1 downregulation in tumor growth and metastatic dissemination in vivo, we injected control and lamin B1 KD MLE12 cells intravenously in nude mice (Fig. 2, H and I). We observed no or very few small nodules in the lungs of mice injected with control MLE12 cells. By contrast, there were numerous metastases with a very large volume in mice injected with lamin B1 KD MLE12 cells (Fig. 2, H and I). Consistently, we observed similar effects on cell migration and invasion in highly metastatic LLC1 cells after lamin B1 depletion (Fig. 2, J and K; and Fig. S2, D and E). Furthermore, we found a significantly increased number of tumor nodules with a large volume in C57BL/6 mice injected intravenously with lamin B1–silenced LLC1 cells compared with controls (Fig. 2, L and M). Thus, lamin B1 depletion promotes EMT, tumor growth, and metastasis.

### Upregulation of the RET proto-oncogene plays a key role in mediating the EMT and malignant phenotype upon lamin B1 loss

**Figure 3.**
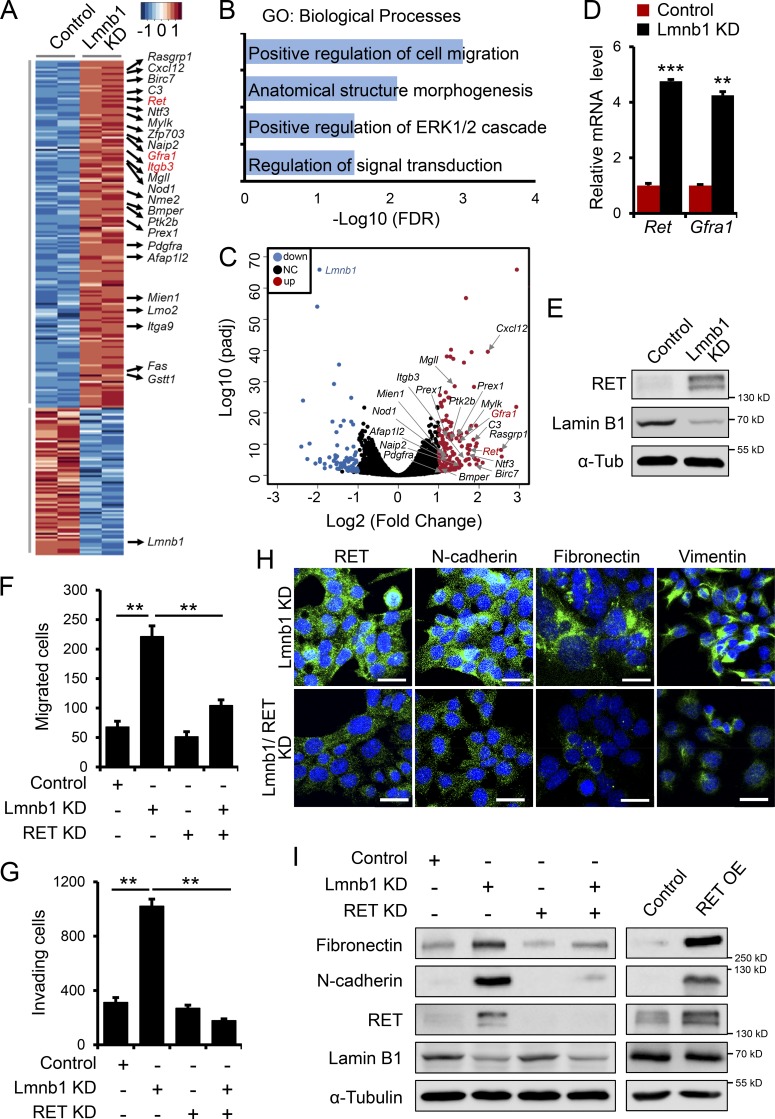
**RET upregulation mediates the EMT phenotype upon lamin B1 loss. (A)** Heat-map representation of RNA-seq analysis of control (*n* = 2) and lamin B1 KD MLE12 (*n* = 2) cells, showing a large number of genes involved in cancer development among the genes upregulated upon lamin B1 loss of function. **(B)** GO terms enriched among genes upregulated upon lamin B1 KD (*n* = 2, fold-change >2; P < 0.05). **(C)** Volcano plot showing log2 fold-change plotted against log10-adjusted P value (padj) for lamin B1 KD versus control MLE12 samples. Red dots represent genes upregulated and blue dots represent genes downregulated (adjusted P < 0.05, fold-change ≤0.5, ≥2) upon lamin B1 loss of function; NC, not changed. Genes involved in cell migration and signaling are indicated. **(D)** qPCR validation of *Ret* and *Gfra1* upregulation upon lamin B1 depletion (*n* = 6). **(E)** Western blot analysis for RET in control and lamin B1 KD MLE12 cells. **(F)** Boyden chamber migration assay with control, lamin B1 KD (Lmnb1 KD), RET KD (RET KD), and lamin B1/RET double-KD MLE12 cells (*n* = 4). **(G)** Boyden chamber invasion assay with control, Lmnb1 KD, RET KD, and lamin B1/RET double-KD LLC1 cells (*n* = 4). **(H)** Immunostaining for mesenchymal markers in lamin B1 KD and lamin B1/RET double-KD MLE12 cells. Cell nuclei were labeled by DAPI. Scale bars, 20 µm. **(I)** Western blot analysis for mesenchymal markers in control, lamin B1 KD, RET KD, and lamin B1/RET double-KD MLE12 cells or control and RET-overexpressing MLE12 cells. Results (D–I) are representative of a minimum of three independent experiments. Statistical analysis was performed using Student’s *t* test with two-tailed distribution. Data are mean ± SEM. **, P < 0.01; ***, P < 0.001. OE, overexpressing; α-Tub, α-tubulin.

To gain insight into the mechanisms mediating the role of lamin B1 in EMT and metastasis we performed RNA sequencing (RNA-seq) from control and lamin B1–depleted MLE12 cells. Consistent with a repressive function of lamin B1 on gene expression (Reddy et al., 2008), we observed a significantly higher number of upregulated genes after lamin B1 depletion (Fig. 3 A and C). Gene Ontology (GO) analysis revealed overrepresentation for GO terms linked to migration and signaling among the genes upregulated upon lamin B1 KD (Fig. 3 B). Particularly interesting was the upregulation of the *Ret* proto-oncogene and its coreceptor *Gfra1* (glial cell line–derived neurotrophic factor family receptor α-1). Quantitative PCR (qPCR) and Western blot analysis confirmed the increased expression of *Ret* and *Gfra1* in lamin B1–depleted MLE12 cells (Fig. 3, D and E; and Fig. S2 F). RET is a member of the receptor tyrosine kinase family that is activated by the glial cell line–derived neurotrophic factor family of ligands. These peptides bind the RET coreceptors from the GFRA family. Ligand binding leads to complex formation between RET and its ligand-bound coreceptors, resulting in kinase activation and stimulation of downstream signaling pathways affecting cell proliferation and migration (Mulligan, 2014). These functional characteristics prompted us to further investigate whether RET upregulation might play a role in mediating EMT and the increased migration upon lamin B1 depletion. Importantly, RET KD led to a significant decrease in the cell migration and invasive capacity of lamin B1–depleted cells (Fig. 3, F and G) and downregulation of mesenchymal markers (Fig. 3, H and I), whereas RET overexpression in MLE12 cells led to a strong increase in mesenchymal marker expression (Fig. 3 I), suggesting that RET might be a key downstream target of lamin B1 in mediating EMT and the increased migratory and invasive phenotype upon lamin B1 loss.

**Figure 4.**
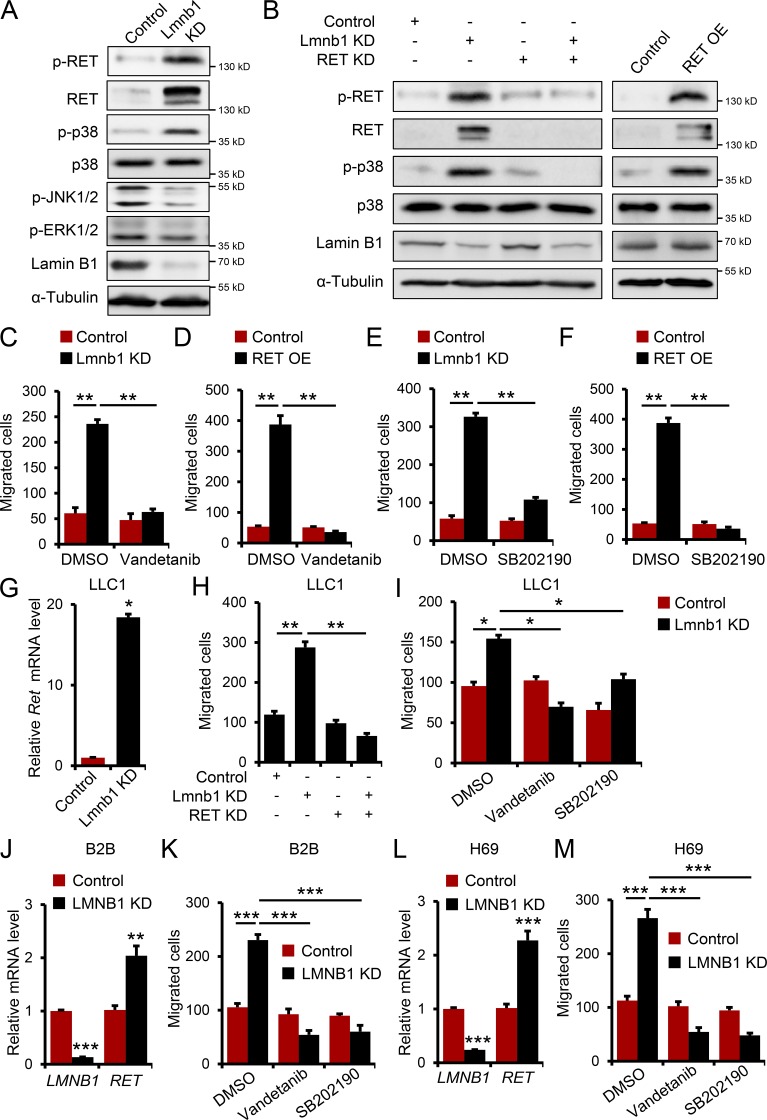
**Inhibition of RET/p38 signaling attenuates the highly migratory phenotype of lamin B1–depleted lung epithelial cells. (A)** Western blot analyses for activated RET (phospho-Y1062-RET), p38, JNK, and ERK1/2 of control and lamin B1 KD MLE12 cells. **(B)** Western blot analyses for RET, p38, activated p-RET, and p-p38 in control, lamin B1 KD, RET KD, and lamin B1/RET double-KD MLE12 cells or control and RET-overexpressing MLE12 cells. **(C and D)** Boyden chamber migration assay with control and lamin B1 KD (C) or control and RET-overexpressing (D) MLE12 cells treated with 20 nM vandetanib (*n* = 4). **(E and F)** Boyden chamber migration assay with control and lamin B1 KD (E) or control and RET-overexpressing (F) MLE12 cells treated with 30 µM SB202190 (*n* = 4). **(G)** Relative expression level of *Ret* in control and lamin B1 KD LLC1 cells (*n* = 6). **(H and I)** Boyden chamber migration assay with control, lamin B1 KD, RET KD , and lamin B1/RET double-KD LLC1 cells (H) or control and lamin B1 KD LLC1 cells treated with 20 nM vandetanib or with 30 µM SB202190 (I; *n* = 4). **(J and L)** Relative expression level of *RET* in control and lamin B1 KD B2B normal human lung bronchial epithelial cells (J) or H69 SCLC cells (L; *n* = 6). **(K and M)** Boyden chamber migration assay with control and lamin B1 KD B2B cells (K) or control and lamin B1 KD H69 cells (M) treated with 20 nM vandetanib or with 30 µM SB202190 (*n* = 4). Results (A–C, E, and G–I) are representative of a minimum of three independent experiments. Data shown in D, F, and J–M are representative of two independent experiments. Statistical analysis was performed using Student’s *t* test with two-tailed distribution. Data are mean ± SEM. *, P < 0.05; **, P < 0.01; *** P < 0.001. OE, overexpressing.

RET activation stimulates numerous signaling pathways affecting cell migration, including the MAP kinases p38, JNK, and ERK. Immunoblot analysis for phosphorylated (p-) and activated RET, p38, JNK1/2, and ERK1/2 revealed a marked increase in RET and p38 activation in lamin B1–depleted MLE12 cells, whereas p-ERK1/2 levels were not changed and p-JNK1/2 levels were even decreased (Fig. 4 A). RET KD in lamin B1–depleted cells led to a significant decrease in activated p-RET, p-p38, whereas RET overexpression led to a strong increase in p-RET and p-p38 (Fig. 4 B), suggesting that activation of the RET/p38 signaling axis can play a role in mediating the increased migratory and invasive phenotype upon lamin B1 loss. Further, treatment of lamin B1–depleted or RET-overexpressing cells with the RET inhibitor vandetanib or the p38 inhibitor SB202190 led to a decrease in the migratory capacity of lamin B1 KD and *Lmnb1*^+/−^ or Ret-overexpressing cells, comparable to control cells (Fig. 4, C–F; and Fig. S2, G and H). In contrast, silencing of lamin A did not have any effect on *Ret* expression and cell migration, suggesting a specific role of lamin B1 in regulating EMT in mouse lung epithelial cells (Fig. S2, I and J). Consistently, we observed similar effects on *Ret* expression and cell migration in lamin B1 KD LLC1 cells, as well as lamin B1 KD LLC1 cells treated with RET and p38 inhibitors, or after RET KD (Fig. 4, G–I). Moreover, similar results were obtained using normal human bronchial epithelium B2B and H69 SCLC cells (Fig. 4, J–M). Thus, activation of RET/p38 signaling is responsible for the highly migratory phenotype of lamin B1–depleted lung epithelial cells.

**Figure 5.**
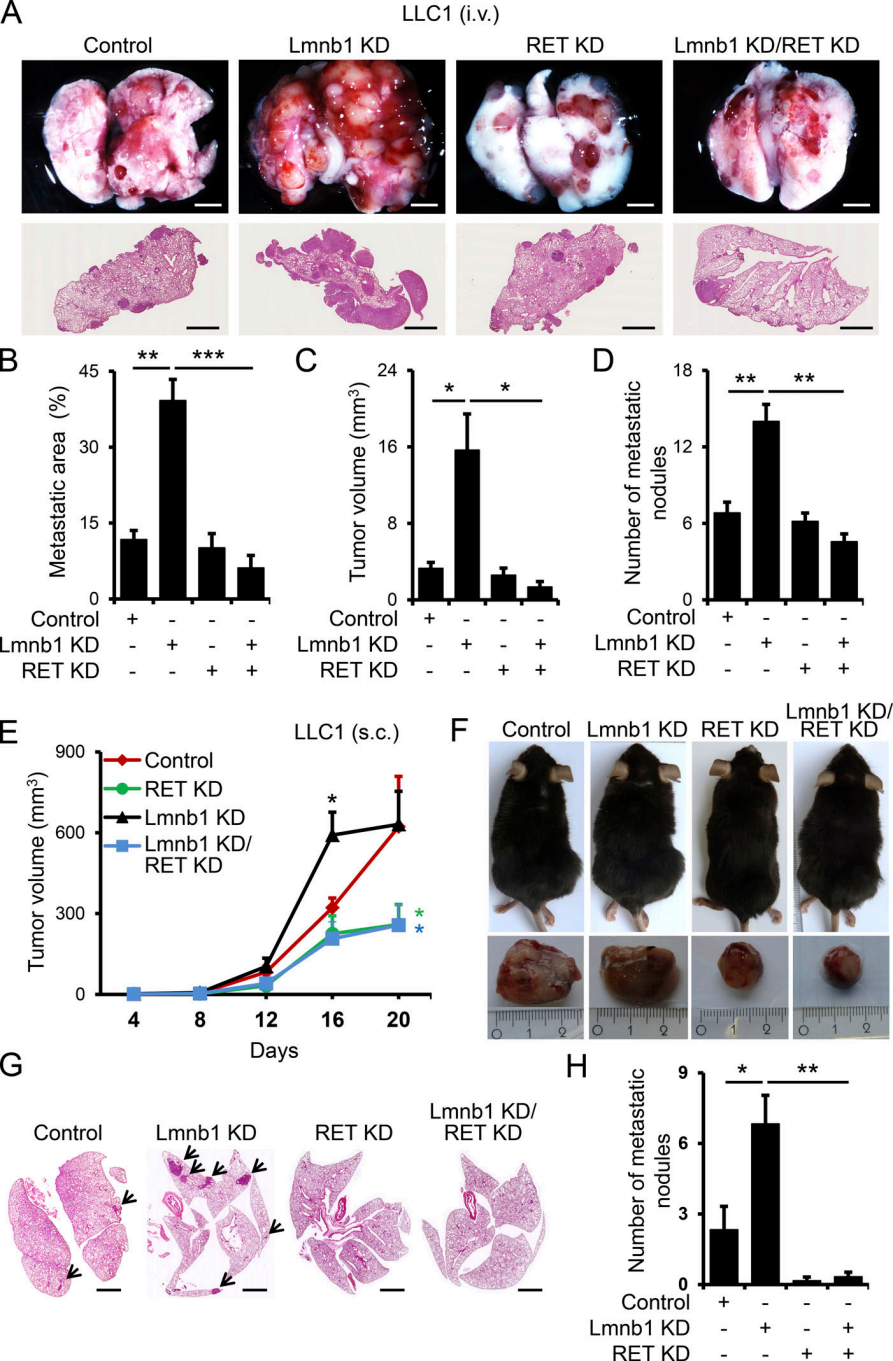
**Lamin B1 KD promotes tumor growth and metastasis through a RET-dependent mechanism. (A–D)** Control, lamin B1 KD, RET KD, and lamin B1/RET double-KD LLC1 cells were injected intravenously into the tail vein of C57BL/6 mice for 20 d (*n* = 6 each). Mice were sacrificed and examined for tumor metastases in the lungs. Macroscopic appearance (A, upper panels) and H&E staining (A, lower panels) of representative lungs. Scale bars, 2 mm. **(B–D)** Quantification of the metastatic area (B), microscopic tumor volume (C), and metastatic nodules (D) of mice injected with control, lamin B1 KD, RET KD, and lamin B1/RET double-KD LLC1 cells. **(E–H)** Control, lamin B1 KD, RET KD, and lamin B1/RET double-KD LLC1 cells were injected subcutaneously into the flanks of C57BL/6 mice for 20 d (*n* = 6). **(E)** Quantification of the macroscopic tumor volume at different days after injection. **(F)** Upper panels: Representative image of the mice at day 20 after injection. Lower panels: Mice were sacrificed and the subcutaneous tumors were removed. **(G and H)** Spontaneous metastasis in lungs. H&E staining of representative lungs (G) and quantification of the number of metastatic nodules in the lungs (H) of mice injected with control, lamin B1 KD, RET KD, and lamin B1/RET double-KD LLC1 cells (*n* = 6). Arrows indicate metastatic nodules. Scale bars, 2 mm. Statistical analysis was performed using Student’s *t* test with two-tailed distribution. Data are mean ± SEM. *, P < 0.05; **, P < 0.01; ***, P < 0.001 (B–E and H).

To examine the role of RET in metastatic dissemination of lamin B1–depleted lung epithelial cells in vivo, we injected control, lamin B1 KD, RET KD, and lamin B1/RET KD LLC1 cells intravenously in C57BL/6 mice (Fig. 5, A–D). Importantly, silencing of RET inhibited the highly increased tumor growth and metastatic ability of lamin B1–depleted cells, evident by the significant decrease in the metastatic area, number of tumor nodules, and tumor volume of mice injected with lamin B1/RET KD compared with lamin B1 KD LLC1 cells (Fig. 5, A–D). To further assess the role of RET and lamin B1 on tumor growth and spontaneous metastasis in vivo, control, lamin B1 KD, RET KD, and lamin B1/RET KD LLC1 cells were injected subcutaneously in C57BL/6 mice (Fig. 5, E–H). The tumor volume of mice transplanted with lamin B1–silenced LLC1 cells was similar to controls at day 20 after injection, although subcutaneous tumors were significantly larger in mice injected with lamin B1 KD LLC1 cells at day 16 (Fig. 5 E). RET depletion in control and lamin B1 KD cells resulted in a significant decrease in tumor volume (Fig. 5, E and F). Importantly, we found a much higher number of spontaneous metastases with larger tumor volume in the lungs of mice injected with lamin B1–depleted LLC1 cells compared with controls, whereas almost no metastasis was observed in mice injected with RET KD and lamin B1/RET KD LLC1 cells (Fig. 5, G and H). Consistent with the RET upregulation upon lamin B1 loss observed in cell culture studies, the metastatic nodules in the lungs of mice injected with lamin B1–depleted LLC1 showed high RET expression (Fig. S2 K). Taken together, these data indicate that RET upregulation plays a key role in mediating the EMT and malignant phenotype upon lamin B1 loss.

### RET levels negatively correlate with lamin B1 levels in lung cancer patients

**Figure 6.**
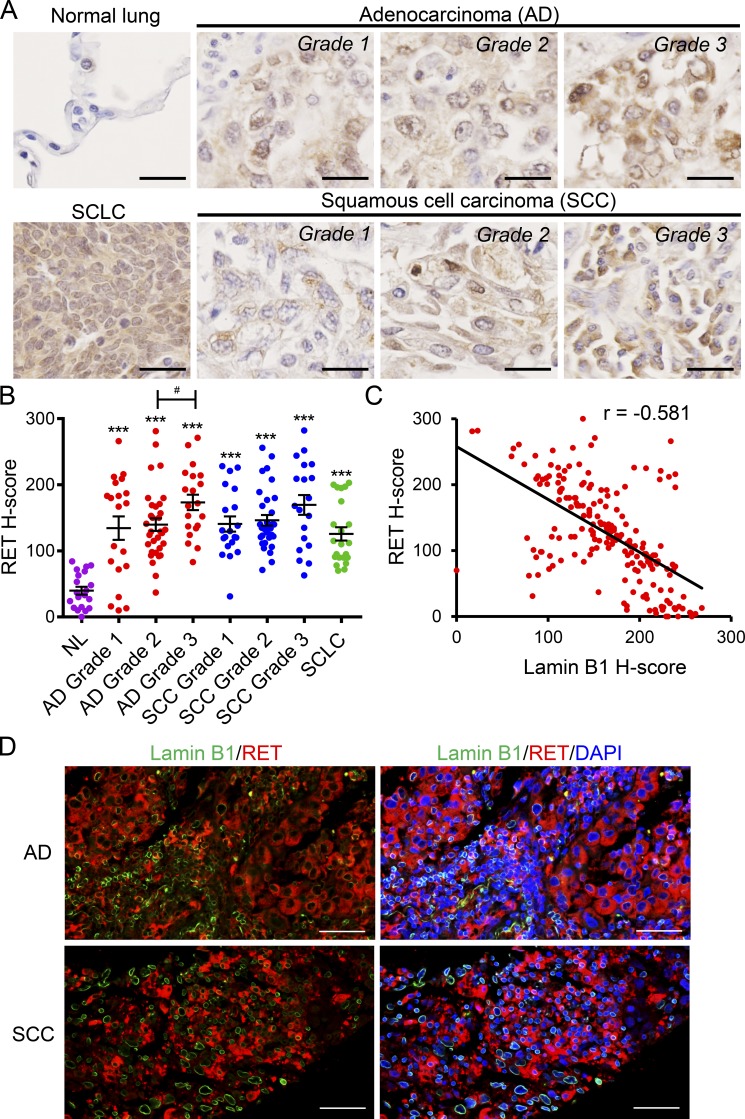
**Lamin B1 and RET expression show inverse correlation in lung cancer patients. (A)** IHC of representative tissue samples from different types and grades of lung tumors, from a tissue microarray, stained with an anti-RET antibody. Scale bars, 25 µm. **(B)** Relative RET staining intensity (H-score) in lung tumors of different types and grades. *n* = 70 adenocarcinoma (20 grade 1, 30 grade 2, 20 grade 3); *n* = 69 SCC (16 grade 1, 33 grade 2, 20 grade 3); *n* = 22 SCLC; and *n* = 20 normal lung tissue (NL). ***, P < 0.001 versus normal lung tissue. #, P < 0.05. AD, adenocarcinoma. **(C)** Correlation between the relative staining intensity for lamin B1 and RET; r, Pearson correlation coefficient. **(D)** Coimmunostaining of representative adenocarcinoma and SCC grade 3 samples with RET and lamin B1 antibodies showing that cells highly positive for lamin B1 express low levels of RET and vice versa. Scale bars, 50 µm.

To analyze whether there is a correlation between lamin B1 and RET levels in lung cancer patients, we analyzed RET expression in a human lung tissue microarray (Fig. 6 A). RET was almost undetectable in normal lung tissue and was significantly upregulated in different types of lung cancer (Fig. 6, A and B). Importantly, the RET expression in the tumors negatively correlated with the lamin B1 expression, supporting a link between lamin B1 loss and RET upregulation in lung cancer patients (Fig. 6, C and D).

### Lamin B1 recruits the EZH1/2 histone methyltransferase to silence gene expression

**Figure 7.**
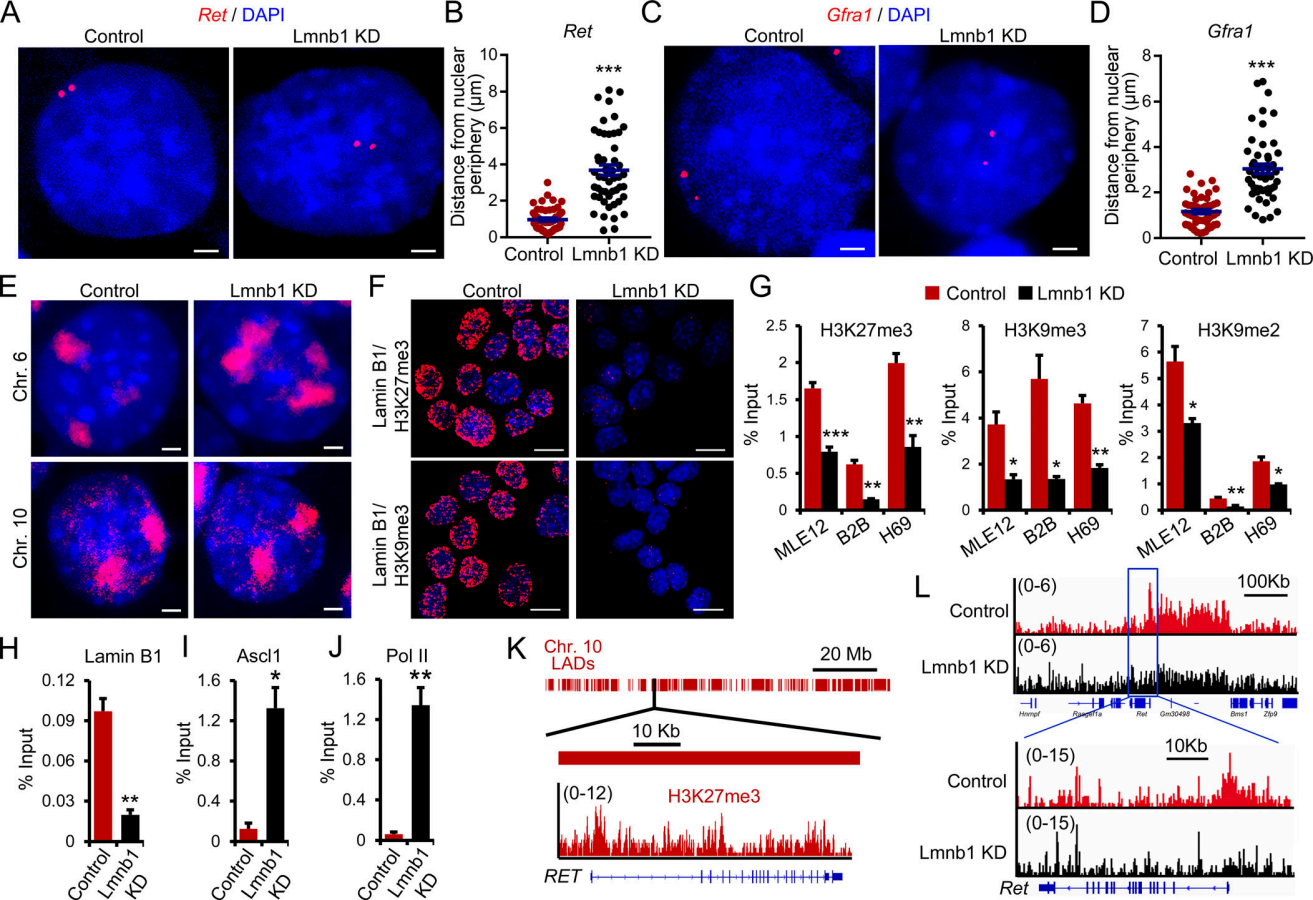
**Lamin B1 loss alters gene positioning and chromatin landscape. (A–D)** Representative DNA FISH images of *Ret* (A) and *Gfra1* (C) genes (red) in control or *Lmnb1* KD MLE12 cells. Cell nuclei were labeled by DAPI. Scale bars, 2 µm. Quantification of the distance of the *Ret* (B) and *Gfra1* (D) genes to the nuclear periphery in individual nuclei of control or Lmnb1 KD MLE12 cells (*n* = 50). **(E)** FISH chromosome painting of Chr. 6 and Chr. 10 in control or Lmnb1 KD MLE12 cells. **(F)** PLA using either lamin B1 and H3K27me3 or lamin B1 and H3K9me3 antibodies. Scale bars, 10 µm. **(G)** ChIP-qPCR analysis of H3K27me3, H3K9me3, and H3K9me2 occupancy at the *Ret* promoter (*n* = 4). **(H–J)** ChIP-qPCR analysis of lamin B1 (H), Ascl1 (I), and Pol II binding (J) at the *Ret* promoter (*n* = 4). **(K)** Schematic representation of the *RET* gene and its localization in LADs (top). Genome tracks of H3K27me3 of human lung tissue showing that the RET promoter is decorated with H3K27me3 mark. **(L)** Genome tracks of H3K27me3 ChIP-seq in control and lamin B1 KD MLE12 cells (*n* = 2). Images in A, C, E, and F are representative of a minimum of two independent experiments. Data shown in B, D, and G–J are mean ± SEM of a minimum of two independent experiments. Statistical analysis was performed using Student’s *t* test with two-tailed distribution. *, P < 0.05; **, P < 0.01; ***, P < 0.001.

**Figure 8.**
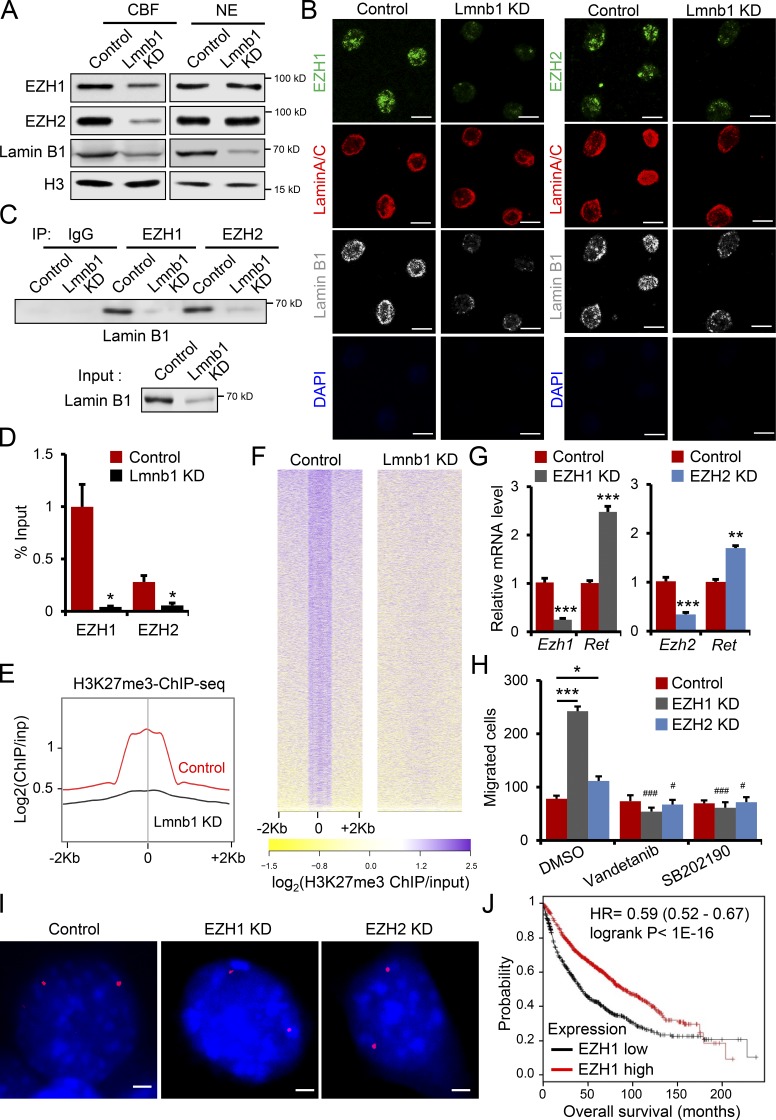
**Lamin B1 recruits EZH1/2 to chromatin to silence *Ret* expression. (A)** Western blot analysis of nuclear extracts (NE) and chromatin bound fraction (CBF) in control and lamin B1 KD MLE12 cells. **(B)** Immunostainings for lamin B1, lamin A/C, and EZH1 or EZH2 of in situ nuclear matrix preparations of control and lamin B1 KD MLE12 cells. DNA content was assessed by DAPI staining, confirming that all chromatin has been removed. Scale bars, 5 µm. **(C)** CoIP of extracts from control and lamin B1 KD cells using control, EZH1, or EZH2 antibodies and detected with anti–lamin B1 antibody. IP, immunoprecipitation. **(D)** ChIP-qPCR analysis of EZH1 and EZH2 binding to the *Ret* promoter (*n* = 4). **(E and F)** Average H3K27me3 ChIP-seq tag intensities (E) and heat map of tag densities in control and lamin B1 KD cells at all H3K27me3 peaks in MLE12 cells (F); inp, input. **(G)** Relative *Ret* expression level in Ezh1 and Ezh2 KD MLE12 cells (*n* = 6). **(H)** Boyden chamber migration assay with control, Ezh1, and Ezh2 KD MLE12 cells treated with DMSO, 20 nM vandetanib, or with 30 µM SB202190 (*n* = 4). Data are mean ± SEM. *, P < 0.05; ***, P < 0.001 *Ezh1* KD or *Ezh2* KD versus control; ###, P < 0.001; #, P < 0.05 versus DMSO. **(I)** Representative DNA FISH images of *Ret* gene loci (red) in control, *Ezh1*, or *Ezh2* knockdown MLE12 cells. Scale bars, 2 µm. **(J)** Overall survival (Kaplan–Meier plot) of lung cancer patients (Győrffy et al., 2013) expressing high versus low levels of EZH1 (probe 203249_at). Results in A are representative of three independent experiments; data in B–D, G, and H are representative of a minimum of two independent experiments. Images in panel I are representative of two independent experiments. Statistical analysis was performed using Student’s *t* test with two-tailed distribution. Data are mean ± SEM. *, P < 0.05; **, P < 0.01; ***, P < 0.001.

To gain further insight into the molecular mechanisms mediating the role of lamin B1 in lung cancer development, metastasis, and gene regulation we first analyzed the spatial gene positioning of the *Ret* (chromosome [Chr.] 6) and its coreceptor *Gfra1* gene loci (Chr. 19) in control and lamin B1–depleted MLE12 cells. Fluorescence in situ hybridization (FISH) analyses revealed that *Ret* and *Gfra1* are found at the nuclear periphery in control cells, whereas in lamin B1–depleted MLE12 cells their association with the nuclear lamina was lost (Fig. 7, A–D). In contrast, *Ret* was found at the nuclear periphery in both control and lamin A–depleted cells, suggesting a specific role of lamin B1 in tethering *Ret* to the nuclear periphery (Fig. S3 A). Interestingly, we observed differences in the distribution of differentially regulated genes among chromosomes (Fig. S3 B); e.g., Chr. 6 showed a higher percentage of upregulated genes in comparison to the percentage of RefSeq genes at this chromosome and Chr. 10 showed a lower percentage (Fig. S3 B). Thus, we investigated the morphology and location of Chr. 6 and Chr. 10 by chromosome painting. In control MLE12 cells, Chr. 6 territories were found near the nuclear lamina and were compact, whereas in lamin B1–depleted cells the nuclear territories occupied by Chr. 6 were larger and more centrally localized (Fig. 7 E). In contrast, no major difference was observed for Chr. 10 (Fig. 7 E). The majority of genes associated with the nuclear lamina are transcriptionally silent and enriched in repressive histone marks such as H3K27me3, H3K9me2, and H3K9me3 (Reddy et al., 2008; Wen et al., 2009). Indeed, proximity ligation assay (PLA) revealed that lamin B1 closely associates with H3K27me3 and H3K9me3 (Fig. 7 F). Importantly, lamin B1 depletion led to significantly decreased levels of H3K27me3, H3K9me2, and H3K9me3 at the *Ret* and *Gfra1* promoters (Fig. 7 G and Fig. S3 C), which were bound by lamin B1 (Fig. 7 H). The transcription factor Ascl1 directly activates *Ret* expression and plays an important role in lung cancer development (Augustyn et al., 2014; Borromeo et al., 2016). Importantly, Ascl1 (Fig. 7 I) and RNA polymerase II binding at the *Ret* promoter was increased in lamin B1–depleted cells (Fig. 7 J). Thus, we analyzed the role of Ascl1 on *Ret* gene positioning and expression in lamin B1–KD MLE12 cells. While FISH analyses revealed that Ret is more centrally localized in both lamin B1–depleted and Ascl1/lamin B1 double-KD cells (Fig. S3 D), Ret expression was significantly downregulated upon Ascl1 depletion in lamin B1–silenced cells (Fig. S3 E), suggesting that not only lack of tethering to the nuclear periphery leads to Ascl1-dependent Ret gene activation. Analysis of lamin B1 and H3K27me3 chromatin immunoprecipitation sequencing (ChIP-seq) data of human lung tissue (Thurman et al., 2012; Shah et al., 2013) revealed that *RET* is located in a large LAD and is decorated by H3K27me3 at its promoter (Fig. 7 K), suggesting similar mechanism of *Ret* regulation in humans. Genome-wide ChIP-seq analysis for H3K27me3 confirmed the decreased levels of H3K27me3 at the *Ret* promoter in lamin B1–depleted lung epithelial cells (Fig. 7 L). The *Ret* promoter is found in a broad H3K27me3 chromatin domain, which showed a global decrease of H3K27me3 upon lamin B1 loss-of-function (Fig. 7 L), suggesting that the PRC2, which catalyzes H3K27 methylation (Cao et al., 2002; Czermin et al., 2002), might play a role in lamin B1–dependent gene regulation. To understand the role of lamin B1 loss on H3K27me3 modification and the role of PRC2 in RET regulation, we first analyzed the expression levels of EZH1 and EZH2, the catalytic subunits of the PRC2 complex. Interestingly, although the global nuclear levels of EZH1/2 did not change (Fig. 8 A and Fig. S4 A), we observed markedly lower levels of EZH1/2 in the chromatin bound fraction of lamin B1–depleted cells (Fig. 8 A). Further, nuclear matrix/intermediate filament preparations demonstrated that EZH factors are retained throughout the nuclear matrix preparations from which all chromatin detectable by DAPI staining has been removed in control MLE12 cells but lost upon lamin B1 depletion (Fig. 8 B). Thus, EZH factors are associated with the nuclear matrix through lamin B1. Moreover, coimmunoprecipitation (coIP) revealed that lamin B1 binds specifically to EZH1 and EZH2 (Fig. 8 C). Consistent with these results, EZH1 and EZH2 were not recruited to the *Ret* promoter in lamin B1–KD cells, whereas in control cells we detected a strong association of both EZH1 and EZH2 to the promoter region of *Ret*, which was also bound by lamin B1 (Fig. 8 D and Fig. 7 H). Importantly, we observed a global decrease in H3K27me3 upon lamin B1 loss-of-function, consistent with the global decrease in chromatin-bound EZH1/2 (Fig. 8, E and F).

Next, we analyzed whether inhibition of EZH1/2 will activate RET expression and phenocopy the increased migratory phenotype of lamin B1–depleted cells. Treatment with the EZH1/2 inhibitor UNC1999 led to a decrease in H3K27me3 levels globally and at the *Ret* promoter and led to an increase in *Ret* expression (Fig. S4, B–D). Importantly, the migration capacity of MLE12 cells was also augmented by UNC1999 application and could be restored to control levels by treatment with the RET inhibitor vandetanib (Fig. S4 E). To further dissect the role of EZH1 and EZH2 in RET regulation and cell migration we depleted EZH1 and EZH2 by lentivirus-driven shRNA-mediated silencing (Fig. 8 G). EZH1 depletion highly increased cell migration and *Ret* expression, whereas EZH2 KD had more modest effects (Fig. 8, G and H). Similarly to the EZH1/2 inhibitor studies, treatment with the RET inhibitor vandetanib reduced migration of EZH1 and EZH2 KD cells to control levels (Fig. 8 H). In addition, treatment with the p38 inhibitor SB202190 led to a decrease in the migratory capacity of EZH1- and EZH2-depleted cells, comparable to control cells (Fig. 8 H). Next, we studied whether EZH1/2 depletion or inhibition affects *Ret* gene localization. We did not observe major changes in *Ret* positioning upon EZH1/2 loss or inhibition (Fig. 8 I and Fig. S4 F), indicating that not only lack of tethering to the nuclear periphery but also loss of EZH1/2 activity is essential for *Ret* gene activation. Consistent with the stronger effects observed on cell migration upon Ezh1 depletion, lung cancer patients expressing low levels of EZH1 had a significantly poorer prognosis than patients with higher EZH1 expression (Fig. 8 J).

### Lamin B1 haploinsufficiency induces spontaneous lung tumor formation and activation of the RET/p38 signaling axis

**Figure 9.**
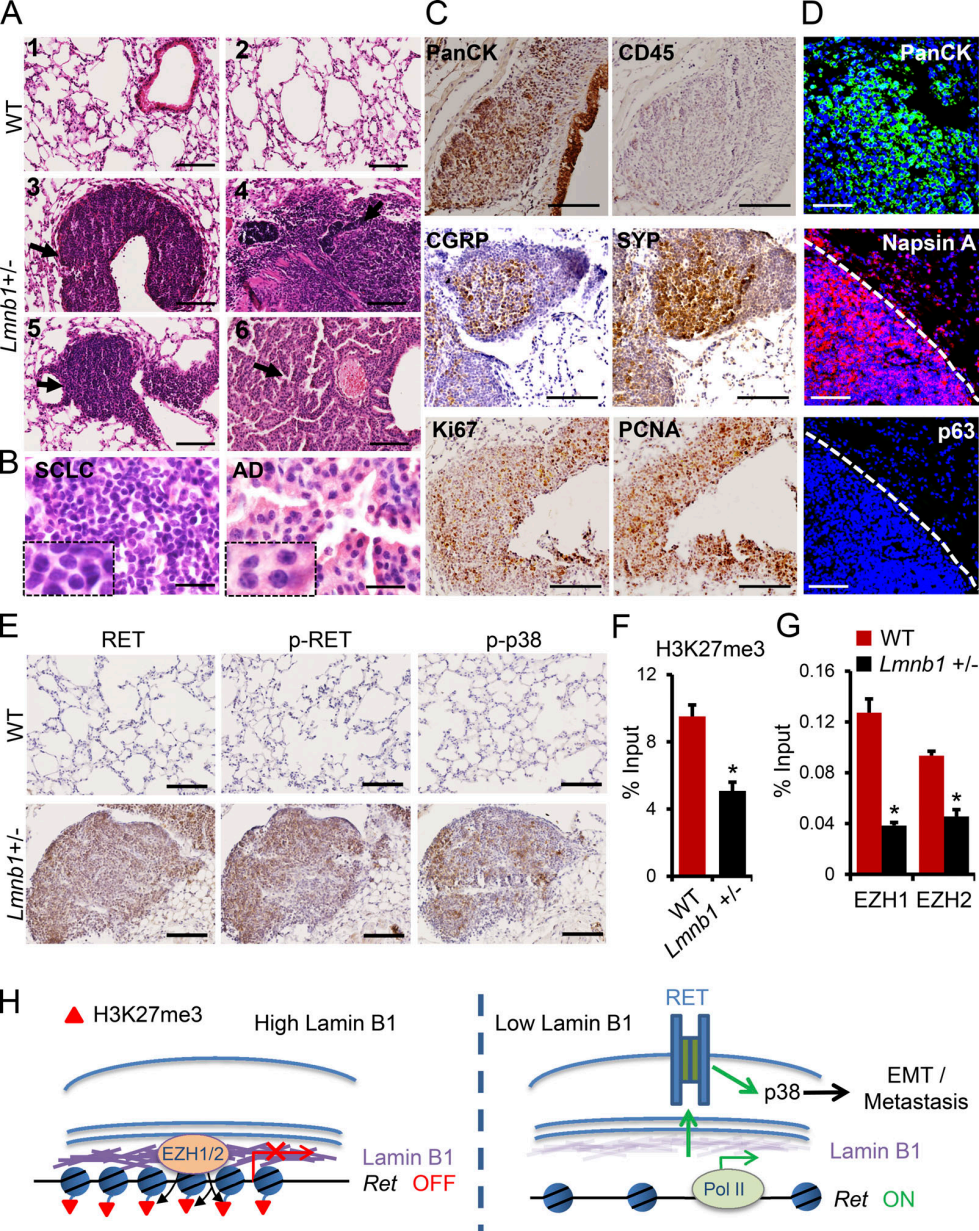
**Lamin B1 haploinsufficiency induces spontaneous lung tumor formation and RET activation. (A)** Histological analysis of representative lung sections of WT (*n* = 27) and *Lmnb1*^+/−^ (*n* = 23) mice at 1.5 yr of age; tumors are indicated by arrows. Scale bars, 100 µm. **(B)** High-magnification images of tumors with SCLC characteristics (left panel) and tumors with adenocarcinoma (AD) characteristics (right panel). Scale bars, 20 µm. **(C)** Immunohistochemical staining of tumors in lungs of *Lmnb1*^+/−^ mice with PanCK, CD45, CGRP, SYP, Ki67, and PCNA. Scale bars, 100 µm. **(D)** Immunostainings of lungs with PanCK, Napsin A, and p63 antibody, confirming that the tumor is adenocarcinoma. Scale bars, 50 µm. **(E)** Immunohistochemical staining of lungs of WT and *Lmnb1*^+/−^ mice with RET, phospho-RET (p-RET Y1062), and phospho-p38 (p-p38 Thr180/Tyr182) antibody. Scale bars, 100 µm. **(F)** ChIP-qPCR analysis of H3K27me3 at the *Ret* promoter in WT and *Lmnb1*^+/−^ lungs (*n* = 4). **(G)** ChIP-qPCR analysis of EZH1 and EZH2 binding to the *Ret* promoter in WT and *Lmnb1*^+/−^ lungs (*n* = 4). **(H)** Model of the role of lamin B1 as a tumor suppressor in lung cancer development. In lung epithelial cells, lamin B1 recruits the EZH1/2 histone methyltransferase to silence the expression of the RET proto-oncogene. Upon lamin B1 loss, recruitment of EZH1/2 to the *Ret* promoter is abolished, leading to an increase in RET expression, tumor growth, and invasiveness. Results in F and G are mean ± SEM of four independent experiments. Statistical analysis was performed using Student’s *t* test with two-tailed distribution. Data are mean ± SEM. *, P < 0.05 (F and G).

We next wanted to corroborate these findings using a genetic approach. To this end, we used mice carrying an *Lmnb1* null allele (Fig. S5 A). In agreement with previous studies (Vergnes et al., 2004; Kim et al., 2011), *Lmnb1*^−/−^ mice died at birth with profound lung abnormalities (Fig. S5 B). *Lmnb1*^+/−^ mice, which showed lower lamin B1 protein levels (Fig. S5, C and D), appeared normal at a young age, although smaller than their littermates. Importantly, older mice had a very high incidence of spontaneous lung tumor formation. 7 out of 12 mice developed spontaneous tumors after 6 mo, whereas 21 out of 23 mice showed tumors by 1.5 yr of age (Fig. 9, A–D; and Fig. S5, E and F). Most of the tumors in *Lmnb1*^+/−^ heterozygous mice showed similar characteristics to SCLC (Fig. 9 A, panels 3–5; and Fig. 9 B, left panel). The cells in these tumors were small with scant cytoplasm and densely packed chromatin, which obscured the nucleoli (Fig. 9 B, left panel). They expressed high levels of cytokeratins (PanCK), markers of epithelial tumors, calcitonin gene–related peptide (CGRP), and synaptophysin (Syp), markers for SCLC of neuroendocrine origin (Meuwissen et al., 2003), but were negative for CD45, a marker for cells of hematopoietic origin (Fig. 9 C). Ki67 and proliferating cell nuclear antigen (PCNA) immunostaining indicated a high proliferative index within these lesions (Fig. 9 C). Apart from tumors with an SCLC phenotype, we also detected tumors in the distal parts of the lungs exhibiting features typical of adenocarcinoma (Fig. 9 A, panel 6; and Fig. 9 B, right panel). Immunoreactivity to cytokeratin, the lung adenocarcinoma marker Napsin A, and negative staining for the SCC marker p63 confirmed that these tumors are adenocarcinomas (Fig. 9 D). These data reveal that the lack of a single functional *Lmnb1* allele is sufficient to efficiently induce spontaneous lung tumor formation. Analyses of other organs revealed tumors in the kidney and the liver. Similarly to the SCLC-like tumors in the lungs of the *Lmnb1*^+/−^ mice (Fig. 9, A–D), the cells in the tumor nodules in livers and kidneys were small with scant cytoplasm, expressed CGRP, Syp, and PanCK, but were negative for CD45 and were found close to blood vessels (Fig. S5 G), suggesting that these might be extrapulmonary metastases rather than primary tumors in these organs. Importantly, the tumors developed in *Lmnb1*^+/−^ mice showed very high RET, activated RET (p-RET) levels, and activated p-p38 levels (Fig. 9 E), supporting a link between lamin B1 deficiency, RET upregulation, and RET/p38 activation in a genetic model of lamin B1 loss-of-function. Next, we analyzed H3K27me3 levels and EZH1/2 recruitment to the *Ret* locus in lungs of WT and *Lmnb1*^+/−^ mice. Consistent with our cell culture studies, we observed a significant decrease in H3K27me3 levels and EZH1/2 occupancy at the *Ret* promoter (Fig. 9, F and G).

In summary, our data suggest that epigenetic derepression of RET, by loss of PRC2 recruitment to chromatin, induces a malignant phenotype in lung epithelial cells with decreased lamin B1 levels (Fig. 9 H).

## Discussion

In this study, we describe a crucial function of lamin B1 in lung cancer development and progression. We show that silencing of lamin B1 or loss of a single lamin B1 allele is sufficient to induce or greatly promote tumor formation. Lamin B1 depletion results in profound changes in the H3K27me3 landscape and promotes EMT, cell migration, tumor growth, and metastasis. We show that epigenetic derepression of RET, by loss of EZH1/2 recruitment to chromatin, plays a key role in mediating the malignant phenotype upon lamin B1 loss. Importantly, RET levels negatively correlate with lamin B1 levels in lung cancer patients, supporting a link between lamin B1 loss and RET upregulation.

We found that lamin B1 levels were reduced in lung cancer patients and that lower lamin B1 expression correlated with higher lung cancer grade. These results were corroborated by our finding that loss of a single functional allele of lamin B1 was sufficient to dramatically increase the occurrence of spontaneous lung tumors in mice. The important role of lamin B1 in cancer development and progression was further supported by cell culture studies and intravenous/subcutaneous injection of LLC1 and MLE12 cells. Lamin B1–depleted MLE12 showed markedly increased anchorage independent growth, one of the hallmarks of malignant transformation (Hanahan and Weinberg, 2011), and dramatically increased tumor growth in vivo. Interestingly, however, proliferation in two-dimensional adherent monolayer cultures was decreased by lamin B1 silencing. Indeed, other studies have shown that anchorage-dependent growth of cells does not correlate to anchorage-independent growth and that nontransformed cells grow faster than transformed cells in high-attachment conditions, in stark contrast to low-attachment conditions (Rotem et al., 2015). Importantly, loss of lamin B1 led to EMT and strongly promoted migration and invasion of mouse and human lung epithelial cells as well as lung cancer cell lines both in culture and in vivo. Lamin disruption may simply affect cell migration and metastasis by increasing the plasticity of nuclear shape, thus facilitating changes in cell form that are required for penetrating restricted spaces, in particular during intravasation of cancer cells from primary tumors into the vasculature and extravasation into metastatic sites (Friedl et al., 2011; Denais et al., 2016). However, RNA-seq after lamin B1 loss of function revealed significant enrichment of genes linked to migration and signaling in the genes upregulated after lamin B1 depletion, suggesting a direct role of lamin B1 in the regulation of cell migration at the gene expression level. We found that increased RET signaling, by upregulation of the RET proto-oncogene and its coreceptor Gfra1, plays a key role in mediating the EMT and malignant phenotype upon lamin B1 loss. RET signaling induces neural crest cell migration by modulating actin dynamics and inducing lamellipodia formation (Fukuda et al., 2002; Asai et al., 2006). Moreover, RET-induced cell migration requires integrins, and β3 integrin (Itgb3) expression correlates with RET-mediated invasion in a tumor xenograft model (Cockburn et al., 2010). Interestingly we observed strong upregulation and activation of several critical components required for RET-mediated migration and invasion upon lamin B1 loss, including Gfra1, Itgb3, and Itga9, suggesting a mechanism by which lamin B1 disruption reinforces RET activation and function in cell migration and invasion.

The RET receptor tyrosine kinase is expressed primarily on neural crest–derived cells and is an important mediator of their growth, differentiation, and migration (Schuchardt et al., 1994; Iwashita et al., 2003). Consistently, RET signaling is deregulated in several neural crest–associated diseases, mostly in neuroendocrine cancers (Mulligan, 2014). Oncogenic *RET* mutations and copy number amplification were also recently found in lung cancer (Kohno et al., 2012; Takeuchi et al., 2012; Yang and Horten, 2014). In line with this, in lamin B1–haploinsufficient mice we observed a high incidence of SCLC, lung tumors of neuroendocrine origin (Sutherland et al., 2011). Importantly, we found very high levels of activated RET (phosphorylated at tyrosine 1062) in the tumors developed by lamin B1–haploinsufficient mice and inverse correlation of lamin B1 and RET expression in lung cancer patients, supporting the notion that RET activation may play a key role in lung cancer development and progression upon lamin B1 loss. Consistent with this, silencing of RET dramatically reduced the highly elevated tumor growth and metastatic ability of lamin B1–depleted cells. RET can activate a variety of signaling pathways (Mulligan, 2014). In particular, phosphorylation of tyrosine 1062 is crucial for activation of the PI3K/AKT, p38, JNK, and ERK–MAPK pathways. We found high levels of activated p38 in lamin B1–depleted lung epithelial cells and in lamin B1–haploinsufficient mice. Inhibition of p38, similarly to RET inhibition, decreased the highly migratory phenotype of lamin B1–depleted lung epithelial cells and lung cancer cells, supporting the notion that activation of the RET/p38 signaling axis mediates the increased migratory and invasive phenotype upon lamin B1 loss.

Changes in the expression of lamins have been reported in various tumor entities; however, the direction of the change appears to be tumor type specific. Thus, while increased levels of lamins were shown in some cancers, such as hepatocellular carcinoma and colorectal and skin cancer (Tilli et al., 2003; Willis et al., 2008; Marshall et al., 2010), a reduction in lamins has been observed in others, including gastric and breast cancer and large B cell lymphoma (Agrelo et al., 2005; Wu et al., 2009; Capo-chichi et al., 2011). These results suggest that, depending on the cellular context, either an increase or a decrease in lamin levels and function can cause an imbalance in lamin-dependent processes that are linked to tumorigenesis, including gene positioning, chromatin organization, and transcription. Indeed, our data show that loss of lamin B1 leads to loss of association of genes upregulated upon lamin B1 depletion with the nuclear periphery. Furthermore, chromosome painting revealed that Chr. 6, which harbors the highest percentage of upregulated genes, is located more centrally and is decondensed in lamin B1–depleted cells. Taken together, these data support an important role of lamin B1 in chromatin positioning and compaction in lung epithelial cells. Further, we found a global decrease in the recruitment of EZH1 and EZH2 to chromatin and reduced H3K27me3 in mouse lung epithelial cells upon lamin B1 depletion. Decreased H3K27me3 might lead to an increase in chromatin accessibility, transcription factor binding, and RNA polymerase recruitment at lamin B1–associated genes, resulting in transcriptional activation. Consistently with this, in lamin B1–depleted cells we observed binding of the Ret promoter by the transcription factor Ascl1, which has been shown to directly activate *Ret* expression and play an important role in lung cancer development (Augustyn et al., 2014; Borromeo et al., 2016). The effects observed upon EZH1 and EZH2 KD suggest that EZH1 might play a more prominent role in mediating the malignant phenotype of lung epithelial cells with decreased lamin B1 levels. Consistently, lung cancer patients expressing low levels of EZH1 had a significantly poorer prognosis than patients with higher EZH1 expression. Indeed, many studies have shown that EZH1- and EZH2-containing complexes have both common and distinct functions (Margueron et al., 2008; Shen et al., 2008). EZH1 maintains H3K27me3 in terminally differentiated cells, whereas EZH2 establishes and maintains H3K27me3 in proliferating cells (Margueron et al., 2008). Consistent with this, we observed markedly higher enrichment of EZH1 compared with EZH2 at the *Ret* promoter. Furthermore, EZH factors also show different biochemical properties. EZH2 has higher catalytic activity toward H3K27me3 than EZH1 and binds to chromatin through JARID2 (Son et al., 2013), whereas EZH1 is able to bind to nucleosomes and induce chromatin compaction also independently of PRC1 recruitment (Margueron et al., 2008), suggesting that EZH1 loss might play a key role in chromatin decompaction upon lamin B1 loss.

Many studies have analyzed the link between gene repositioning and gene activity. While in some cases gene repositioning leads to changes in gene activity, in other cases it does not (reviewed in Shachar and Misteli, 2017). The loss of lamin B1 was associated with *Ret* repositioning from the nuclear lamina toward the center, concomitant with Ret upregulation. At the same time, KD of neither *Ezh1* nor *Ezh2* led to *Ret* gene repositioning, but elicited a significant increase in RET expression and migratory capacity of MLE12 cells, suggesting that not only lack of tethering to the nuclear periphery but also loss of Ezh1/2 binding to chromatin is essential for *Ret* gene activation. An intriguing question that needs further investigation concerns the upstream mechanisms leading to lamin B1 downregulation in lung cancer patients. Analysis of TCGA data indicates that in human lung tumors LMNB1 is rarely affected by point mutations but lies within a relatively large region of recurrent deletion (data not shown), which may contribute to a decrease in LMNB1 levels in lung cancer. Epigenetic mechanisms, such as promoter hypermethylation, may also play a role. For example, CpG island promoter hypermethylation leads to lamin A/C silencing in lymphoma and neuroblastoma cells (Agrelo et al., 2005; Rauschert et al., 2017). Since *LMNB1* has a TATA-less CpG island-associated promoter, promoter hypermethylation might be also responsible for *LMNB1* downregulation.

Similarly to lamin B1, the role of PRC2 complex in cancer development is complex and cell-type specific. Both gain and loss of PRC2 function, as a result of mutations in several PRC2 components, have been linked to cancer initiation, progression, and metastasis (Dawson and Kouzarides, 2012; Conway et al., 2015). In addition, the role of PRC2 in EMT appears also to be cell-type specific (Tiwari et al., 2013; Cardenas et al., 2016). Thus, targeting molecules downstream of the lamin B1–EZH1/2 axis might be a better therapeutic strategy. We found that RET upregulation and activation of the RET/p38 signaling axis plays a key role in the malignant phenotype upon lamin B1 depletion. Importantly, treatment of lung epithelial cells with RET and p38 inhibitors completely reversed the highly migratory and invasive phenotype after lamin B1 loss and EZH1/2 inhibition or depletion. The benefit of RET inhibitors (cabozantinib, vandetanib, and lenvatinib), in terms of response and median progression-free survival of NSCLC patients with activating RET rearrangements and mutations has been demonstrated in several clinical trials (reviewed in Mendoza, 2018). Ongoing early-phase clinical trials using the highly potent and selective RET kinase inhibitors BLU-667 and LOXO-292 have generated great interest, given the high level of response and very mild toxicity profiles (Subbiah et al., 2018a,b). Since our findings revealed decreased expression of lamin B1 in the majority of lung carcinoma patients, targeting of RET, or p38 signaling, might be a valuable therapeutic strategy for a significant fraction of human lung tumors caused by perturbation of the lamin B1–EZH2 axis.

## Materials and methods

### Animal experiments

All animal experiments were done in accordance with the institutional guidelines and are covered in an approved animal experimental protocol by the Committee for Animal Rights Protection of the State of Hessen (Regierungspraesidium Darmstadt, Germany; Experimental protocol reference numbers V54–19 c 20/15–B2/363 and V54-19c20/15-B2/1113).

### Mouse lines

The Lmnb1tm1a (EUCOMM)Wtsi mouse line was obtained from Wellcome Sanger Institute, Genome Research Limited. Lmnb1tm1a mice were backcrossed to C57BL/6 mice for six generations before use in these studies.

### Tumor cell transplantation and analysis of metastatic growth

Female C57/BL6 and BALB/c nu/nu mice were purchased from Charles River and kept under pathogen-free conditions. For induction of experimental metastasis, 10^6^ WT, control, or lamin B1, RET, and lamin B1/RET shRNA-expressing LLC1 and MLE12 cells were injected intravenously into 7–8-wk-old C57BL/6 and BALB/c nu/nu mice, respectively. After 20 d (LLC1 cells) or 24 d (MLE12 cells), the mice were sacrificed, and the lungs were isolated, imaged, and fixed with 3.7% paraformaldehyde and embedded into paraffin. 5-µm-thick paraffin sections were stained with H&E.

For subcutaneous transplantation, 10^6^ control or lamin B1, RET, and lamin B1/RET shRNA-expressing LLC1 cells were injected into the flanks of C57BL/6 mice (Charles River). After 20 d, the mice were sacrificed and the subcutaneous tumors, and lungs were isolated.

Metastatic area was defined as the percentage of lung area occupied by metastatic tumor, measured by ImageJ (National Institutes of Health). Tumor volumes and number of tumor nodules in the lung were quantitated from H&E-stained lung sections using ImageJ.

### Histology

Lungs were perfused, dissected, and fixed with 3.7% paraformaldehyde at room temperature for 1 d, followed by three washes with PBS. Tissues were dehydrated by incubation in a series of ethanol solutions with increasing concentrations. Ethanol was replaced by xylene and paraffin followed by embedding in paraffin. H&E staining was performed according to the manufacturer’s instruction (GHS116, HT-110216; Sigma-Aldrich). Representative images of histological analysis of mice with the same genotype are presented.

### Antibodies, chemicals, and plasmids

Detailed information regarding the antibodies, chemicals, and plasmids used in this study is provided in Table S1.

### Immunohistochemistry (IHC) and immunofluorescence staining

For immunohistochemical staining on paraffin sections, slides were heated at 55°C for 10 min and submerged into xylene (Sigma-Aldrich), followed by serial deparaffinization steps (2× xylene, 100% ethanol, 80% ethanol, 50% ethanol; each 5 min). Antigen retrieval was done by microwave boiling in 10 mM citrate buffer for 10 min. The subsequent steps were done with the VECTASTAIN Universal Quick HRP Kit (PK-7800; Vector Laboratories) following the manufacturer’s instructions. Staining was developed with DAB Peroxidase (HRP) Substrate Kit (SK-4100; Vector Laboratories). Images were acquired by NanoZoomer 2.0-HT (Hamamatsu) or Axio Scan.Z1 (Zeiss) and analyzed by NDP.view2 Viewing software (Hamamatsu) or ZEN 2.3 SP1 (Zeiss), respectively.

For immunofluorescence staining on paraffin sections, slides were deparaffinized and antigen retrieval steps were performed as described for IHC staining on paraffin sections. Slides were then blocked with 10% FBS with 0.5% Triton X-100 (Sigma-Aldrich) in PBS for 1 h and incubated with primary antibodies in blocking buffer at 4°C overnight. On the next day, slides were washed and secondary antibodies were added for 2 h. Slides were then washed and mounted in anti-fade mounting medium.

For immunofluorescence staining of cultured cells, cells were counted and seeded on coverslips in 24-well plates. Next, cells were washed with PBS and fixed with 3.7% formaldehyde for 10 min, followed by the same blocking and staining procedures as described for immunofluorescence staining on paraffin sections. All of the fluorescence images were acquired using LSM 700 laser scanning microscope (Carl Zeiss Micro Imaging) with a 25×, 40×, or 63× objective and analyzed with ImageJ (National Institutes of Health) or Zeiss ZEN Microscope Software (Carl Zeiss Micro Imaging).

### Human tissue microarray immunostaining and analysis

Commercially available high-density lung cancer tissue arrays, containing 168 cases of different types and grades of lung cancer (grade 1–3), were purchased from US Biomax, Inc. (LC2085c). Deparaffinization, antigen retrieval, blocking, staining, and image acquisition were the same as described for IHC staining on paraffin sections. For lamin B1 and lamin A/C staining, only the nuclear staining signal of cancer cells excluding nonmalignant stromal cells and immune cells was quantified. The quantification of the signal was performed by measurement of signal intensity and percentage of stained cells. The intensity of the staining signal was semiquantitatively graded on a scale from 0 to 3 (lowest to highest). H-score was calculated by multiplying the staining intensity by the percentage of stained cells. The staining score ranges from 0 to 300.

### Cell culture, generation of stable cell lines, and CRISPR/Cas9–*Lmnb1*^+/−^ MLE12 cells

MLE12, LLC1, BEAS-2B (B2B), NCI-H69 (H69), and HEK293T cells were purchased from the American Type Culture Collection (CRL-2110, CRL-1642, CRL-9609, HTB-119, and CRL-3216). MLE12 cells were cultured in DMEM/F12 medium supplemented with 10% FBS and penicillin/streptomycin/glutamine (PSG). HEK293T and LLC1 cells were cultured in DMEM supplemented with 10% FBS and PSG. B2B and H69 cells were cultured in Roswell Park Memorial Institute 1640 supplemented with 10% FBS and PSG. For the generation of stable cell lines, 0.5 × 10^6^ HEK293T cells were plated on a 6-well plate and transfected with 2 µg of plasmids containing shRNAs for lamin B1, lamin A/C, RET, Ascl1, EZH1, EZH2, or control and RET-overexpressing constructs, along with packaging plasmids, obtained from The RNAi Consortium shRNA library using FuGENE (Roche) transfection reagent. 48 h after transfection, cells were transduced with viral supernatant. 48 h after transduction, cells were selected with 10 µg/ml puromycin for two passages and selected cells were maintained with 2 µg/ml puromycin as stable cell lines. For generation of *Lmnb1*^+/−^ MLE12 cells by CRISPR/Cas9-mediated gene targeting, a combination of two guide RNAs (gRNAs) was used as follows: gRNA-1: 5′-CAC​CGA​AAC​TCT​AAG​GAT​GCG​GCG​C-3′ and 5′-AAA​CGC​GCC​GCA​TCC​TTA​GAG​TTT​C-3′; gRNA-2: 5′-CAC​CGA​GAG​GCT​CTC​GAT​CCT​CAT​C-3′ and 5′- AAA​CGA​TGA​GGA​TCG​AGA​GCC​TCT​C-3′. pSpCas9(BB)-2A-Puro (PX459) V2.0, a gift from Feng Zhang (Broad Institute, Cambridge, MA; Addgene plasmid no. 62988; Ran et al., 2013) was digested and ligated with annealed gRNAs into the recombinant plasmid pSpCas9(BB)-2A-Puro-gRNA-1 and pSpCas9(BB)-2A-Puro-gRNA-2. Both plasmids were transfected into MLE12 cells using Lipofectamine 2000 according to the manual. Positive cells were selected using puromycin (2 µg/ml) for 3 d before clonal expansion and single clone selection. All cell lines used in the present study were free from mycoplasma contamination.

### Scratch wound assay

LLC1 and MLE12 cells expressing control or lamin B1 targeting shRNA were seeded in a 6-well plate. After reaching 100% confluence, scratches were made with pipette tips and images were acquired immediately after scratching, as well as 12 h and 24 h later.

### Boyden chamber migration and invasion assay

Boyden chamber inserts were placed on 24-well plates containing 10% FBS medium. 10^5^ MLE12 cells, 5 × 10^4^ LLC1 cells, 1.2 × 10^5^ B2B cells, or 2 × 10^5^ H69 cells were seeded on top of the insert in a 0% FBS medium. 5 h later (for H69 cells, 24 h later), the insert membranes were fixed with 3.7% formaldehyde for 10 min and washed three times with PBS. The upper surface of the membranes was cleaned by wiping with a piece of wet paper. Membranes were then stained with crystal violet for 10 min. Images of randomly selected areas were acquired with a 20× objective and the number of cells that had migrated to the lower surface of the membrane was quantified using ImageJ (National Institutes of Health). The in vitro Matrigel invasion assay was essentially similar to the cell migration assay described above, except that the membrane filter was precoated with 50 µl diluted Matrigel (diluted with serum-free culture medium, final working concentration 1 mg/ml) for 2 h before seeding the cells. 24 h later, the insert membranes were fixed.

### Cell proliferation and soft agar colony formation assay

Cell proliferation was measured using CellTiter 96 AQueous One Solution Cell Proliferation Assay (MTS; 3-(4,5-dimethylthiazol-2-yl)-5-(3-carboxymethoxyphenyl)-2-(4-sulfophenyl)-2H-tetrazolium, inner salt) according to the manufacturer’s instructions. Absorbance, which is proportional to the number of living cells in culture, was measured at 490 nm. For soft agar colony formation assay, control and Lmnb1 KD MLE12 cells were suspended in complete DMEM/F12 medium containing 0.3% low melting agarose and plated onto solidified 0.6% agarose in complete DMEM/F12 medium in 12-well culture plates at a density of 5,000 cells per well. After 14 d, the colonies were stained with 0.005% Crystal Violet.

### Immunoprecipitation and immunoblotting

For coIPs, transfected cells were lysed in coIP buffer (50 mM Tris pH 7.5, 100 mM NaCl, 15 mM EGTA, 0.1% Triton X-100, and protease inhibitors SET-I; Sigma-Aldrich), sonicated for 10 s (Bandelin Sonopuls), and the extracts were clarified. The lysates were then incubated with the indicated antibodies overnight at 4°C followed by 3 h incubation with Protein-A/G-Sepharose beads (GE Healthcare). Immunoprecipitates were washed five times in coIP buffer, dissolved in 2× SDS-PAGE sample buffer, and subjected to standard Western blot analysis.

### PLA

PLA was performed using Duolink In Situ Orange Starter Kit Goat/Rabbit (DUO92106; Sigma-Aldrich), following the manufacturer’s instructions. Images were captured using LSM 700 (Carl Zeiss Micro Imaging) with a 63× objective and analyzed with ImageJ (National Institutes of Health).

### DNA FISH

DNA FISH probes for *Ret* and *Gfra1* were labeled with digoxigenin by a Nick Translation kit (Roche) according to the manufacturer’s protocol using bacterial artificial chromosome DNA clones: Ret (RP23-98B12; Thermo Fisher Scientific) and Gfra1 (RP23-180P13; Thermo Fisher Scientific). Whole-chromosome painting probes for mouse Chr. 6 and Chr. 10 were purchased from MetaSystems Probes. For DNA FISH, cells were grown on coverslips, fixed with 4% formaldehyde for 10 min, and permeabilized with 0.5% Triton X-100 for 10 min. Further, cells were incubated with 20% glycerol/PBS for 60 min followed by treatment with 0.1 N HCl for 20 min and washes with 2× saline-sodium citrate (SSC) buffer (1× SSC: 0.15 M NaCl and 0.015 M sodium citrate, pH 7.0) for 5 min and 50% formamide/2× SSC for 30 min. Next, cells were hybridized with a labeled DNA probe for 24 h at 37°C. Probes were detected by anti–digoxigenin-rhodamine, Fab fragments (Roche), and cell nuclei were counterstained with DAPI. Images were acquired using LSM 700 (Carl Zeiss Micro Imaging) with a 63× objective and analyzed with ImageJ (National Institutes of Health) or Zeiss ZEN Microscope Software (Carl Zeiss Micro Imaging).

### RNA isolation, RT-PCR, and real-time PCR

RNA was isolated using the TRIzol RNA Isolation Reagent (Invitrogen). For real-time PCR analysis, cDNA was synthesized with the High Capacity cDNA Reverse Transcription Kit (Applied Biosystems), and real-time PCR was performed using the SYBR Green PCR master mix (Applied Biosystems). Cycle numbers were normalized to these of α-tubulin (Tuba1a).

RT-qPCR primers used in this study were as follows: mouse Lmnb1: forward (5′-AGA​TCA​GGG​ACC​AGA​TGC​AG-3′), reverse (5′-GAA​GGG​CTT​GGA​GAG​AGC​TT-3′); human Lmnb1: forward (5′-GAC​CAG​CTG​CTC​CTC​AAC​TAT​G-3′), reverse (5′-ATT​CTC​GAA​GCT​TGA​TCT​GGG​C-3′); mouse Lmna: forward (5′- GGA​AGT​CGA​TGA​AGA​GGG​AAA​G-3′), reverse (5′-TTT​AGG​GTG​AAC​TTC​GGT​GG-3′); mouse Tuba1a: forward (5′-CCG​CGA​AGC​AGC​AAC​CAT-3′), reverse (5′-CCA​GGT​CTA​CGA​ACA​CTG​CC-3′); human Tuba1a: forward (5′-GAA​GCA​GCA​ACC​ATG​CGT​GA-3′), reverse (5′-TCT​CCT​CCC​CCA​ATG​GTC​TT-3′); mouse Ret: forward (5′-ACA​CGG​CTG​CAT​GAG​AAT​GA-3′), reverse (5′-GGA​AAC​CAC​CAT​TGC​GGA​TG-3′); human Ret: forward (5′-GAA​GGC​GAC​GTC​CGG​TG-3′), reverse (5′-TAG​AGG​CCC​AAT​GCC​ACT​TT-3′); mouse Ascl1: forward (5′-CCC​TCT​TAG​CCC​AGA​GGA​AC-3′), reverse (5′-TGC​CAT​CCT​GCT​TCC​AAA​GTC-3′); mouse Ezh1: forward (5′-TCC​ATG​AGG​AAA​ATG​GAT​ATA​GCA-3′), reverse (5′-TCC​CAT​ATT​TGC​CTG​GAG​CC-3′); mouse Ezh2: forward (5′-ACT​GCT​TCC​TAC​ATC​CCT​TCC-3′), reverse (5′-ACG​CTC​AGC​AGT​AAG​AGC​AG-3′); ChIP mouse Ret: forward (5′-GAA​AGA​GGG​ACA​GAG​AGC​CT-3′), reverse (5′-GAC​AAC​GGT​AGC​AGG​TCT​CT-3′); ChIP human Ret: forward (5′-TAG​CCG​CAG​TCC​CTC​CAG-3′), reverse (5′-CCC​ACG​GCA​AAC​AGA​AAG​G-3′); ChIP mouse Gfra1: forward (5′-GAC​CCG​CTT​TTA​GGG​GTT​CA-3′), reverse (5′-CTT​CAG​CAC​TCT​GGG​CTC​TC-3′); ChIP human Gfra1: forward (5′-TGC​GGT​AAT​CTT​CGA​GAG​CT-3′), reverse (5′-GAA​CAG​GAG​CAG​GCC​GAG-3′). For RNA-seq, the RNA was isolated using RNeasy Microarray kit (73304; Qiagen).

### RNA-seq data analysis

Raw RNA-seq reads were trimmed with Reaper (http://www.ebi.ac.uk/~stijn/reaper/reaper.html) using the following parameters: -geom no-bc -3pa -clean-length 20 -qqq-check 53/10 -trim-length 150 –nozip. Trimmed reads were mapped to the reference genome mm10 (UCSC assembly) using STAR software (Dobin et al., 2013) with parameters (--outFilterMismatchNoverLmax 0.1 --chimSegmentMin 18 --chimScoreMin 12). Differential expression (Log2FC [fold change] ≥1, false discovery rate [FDR] <0.05, BaseMean ≥2 for upregulated genes, Log2FC ≤ −1, FDR <0.05, BaseMean ≥2 for downregulated genes) was quantified and normalized by the use of DESEq2. GO analysis was performed using DAVID Bioinformatics Resources 6.8; adjusted P value (FDR) for the GO terms was calculated in R using the Benjamini-Hochberg method (Benjamini and Hochberg, 1995). The percentage of upregulated genes per chromosome was calculated by dividing the number of genes upregulated upon Lmnb1 KD in MLE12 cells at a specific chromosome to the total number of upregulated genes. To determine the number of genes per chromosome, a custom script in bash was developed. Briefly, the coordinate of mm10 transcripts of Refseq database were obtained by using (analyzeRepeats.pl rna mm10 -strand both -count genes) and annotated by annotatePeaks.pl (default settings). Single genes per chomosomes were substracted with the help of an R script mat_u <- mat[!duplicated(mat$gene_name),] followed by mat_u <- subset(mat_u, chr==n), where n is each chromosome identifier.

### In situ nuclear matrix extraction

Cells on the coverslips were washed and incubated with cytoskeletal buffer (10 mM PIPES, pH 6.8, 100 mM NaCl, 300 mM sucrose, 1 mM EGTA, and 1 mM MgCl_2_) supplemented with protease inhibitors and 0.5% Triton X-100 at room temperature for 3 min. Chromatin was digested with DNase I (50 ng/µl) in DNase I buffer (10 mM Tris-HCl, pH 7.5, 2.5 mM MgCl_2_, and 0.5 mM CaCl_2_) at room temperature for 30 min. The coverslips were washed with PBS, followed by immunostaining.

### ChIP and ChIP sequencing

5 × 10^6^ control and lamin B1 KD MLE12 cells were fixed with 1% formaldehyde for 10 min at room temperature. Fixation was quenched with 125 mM glycine followed by washes with cold PBS for 3 × 10min. Cells were further lysed in 600 µl of ice-cold L1 lysis buffer (Tris pH 8.0, 50 mM; EDTA pH 8, 2 mM; NP-40 0.1%; and glycerol 10%) for 5 min and nuclei were spun down. Next, nuclei were resuspended in 600 µl L2 lysis buffer (SDS 1%; EDTA pH 8, 5 mM; and Tris pH 8, 50 mM) and DNA was sheared by sonication. Extracts were precleared with 80 µl protein G beads for 1 h followed by incubation with 10 µg primary antibody overnight at 4°C and binding to 30 µl BSA-coated protein G beads. Immunoprecipitates were washed two times with NaCl-washing buffer (0.1% SDS, NP-40 1%, 2 mM EDTA, 500 mM NaCl, and 20 mM Tris pH8), followed by two washes with LiCl-washing buffer (0.1% SDS, 1% NP-40, 2 mM EDTA, 500 mM LiCl, and 20 mM Tris pH 8) and eluted with extraction buffer (Tris pH 8.0, 20 mM, EDTA pH 8, 2 mM, 2% SDS). Cross-linking was reverted by incubation overnight at 65°C. DNA was purified using Qiagen mini-elute PCR purification kit (28004). Immunoprecipitated DNA was either used for qPCR analysis or sequenced on NextSeq500 instrument (Illumina).

### ChIP-seq data analysis

ChIP-seq reads were mapped to the mouse reference genome mm10 (UCSC assembly) using the default settings of Bowtie2 (Langmead and Salzberg, 2012). The PCR duplicates were removed with MarkDuplicates.jar from picard-tools-1.119. Peaks in H3K27me3-ChIP-seq were called using findPeaks from homer (-region -size 1000 -minDist 10000 -style histone) using the Input as control sample. The common peaks between replicates (*n* = 2) were obtained by the use of bedtools intersect (-wa). Distribution of the H3K27me3-ChIP mapped reads from control and lamin B1 KD MLE12 cells at all peaks in MLE12 was performed using ngs.plot (Shen et al., 2014) with mm10. The BAM files were normalized to reads per genome coverage by the use of BamCoverage from deepTools2 (Ramírez et al., 2014; -b 20 -smooth 40 -e 150 --normalizeTo1x 2652783500).

### Statistical analysis

Results from quantitative analyses are presented as mean ± SEM. Statistical analysis was performed using Student’s *t* test with two-tailed distribution. Sample sizes were determined based on previous experience with analogous experiments. For in vivo experiments, animals that unexpectedly died were excluded from the analysis. Statistical significance was defined as follows: *, P < 0.05; **, P < 0.01; ***, P < 0.001; or #, P < 0.05; ##, P < 0.01; ###, P < 0.001 in Fig. 1 C, Fig. 6 B, Fig. 8 H, and Fig. S1 B.

### Data availability

Sequencing data (ChIP-seq and RNA-seq) have been deposited in the Gene Expression Omnibus under accession number GSE94681. All data supporting the findings of this study are included in the manuscript and are available from the corresponding author on request.

### Online supplemental material

Fig. S1 shows immunohistochemical staining with a second lamin B1 antibody on human lung tissue microarrays validating the findings presented in Fig. 1. Fig. S1 also shows high lamin B1 levels in nonmalignant stroma cells. In addition, abnormal nuclear shape and lamin B1 localization in LLC1 cells are presented in Fig. S1. Fig. S2 shows validation of the specific role of lamin B1 in *Ret* gene regulation, cell migration, and EMT in MLE12 cells using shRNA against *Lmna*, a second shRNA against *Lmnb1*, as well as *Lmnb1*^+/−^ MLE12 cells generated by CRISPR/Cas9 gene editing. Fig. S3 presents FISH analysis of Lmna KD, Lmnb1 KD, and Lmnb1/Ascl1 double-KD MLE12, as well as chromatin changes at the *Gfra1* locus. Fig. S4 shows the effects of EZH1/2 inhibition on *Ret* gene positioning and expression or cell migration. Fig. S5 presents further characterization of the lung defects in *Lmnb1*^+/−^ and *Lmnb1*^−/−^ mice.

## Supplementary Material

Supplemental Materials (PDF)
