# Supplementary material for: Lamin B1 loss promotes lung cancer development and metastasis by epigenetic derepression of RET
Source: J Exp Med. 2019 Apr 23;216(6):1377–95. doi: 10.1084/jem.20181394 (PMC6547854; doi:10.1084/jem.20181394)
Supplement: Supplemental Materials (PDF) [file JEM_20181394_sm.pdf]

Supplemental material

Jia et al., https://doi.org/10.1084/jem.20181394

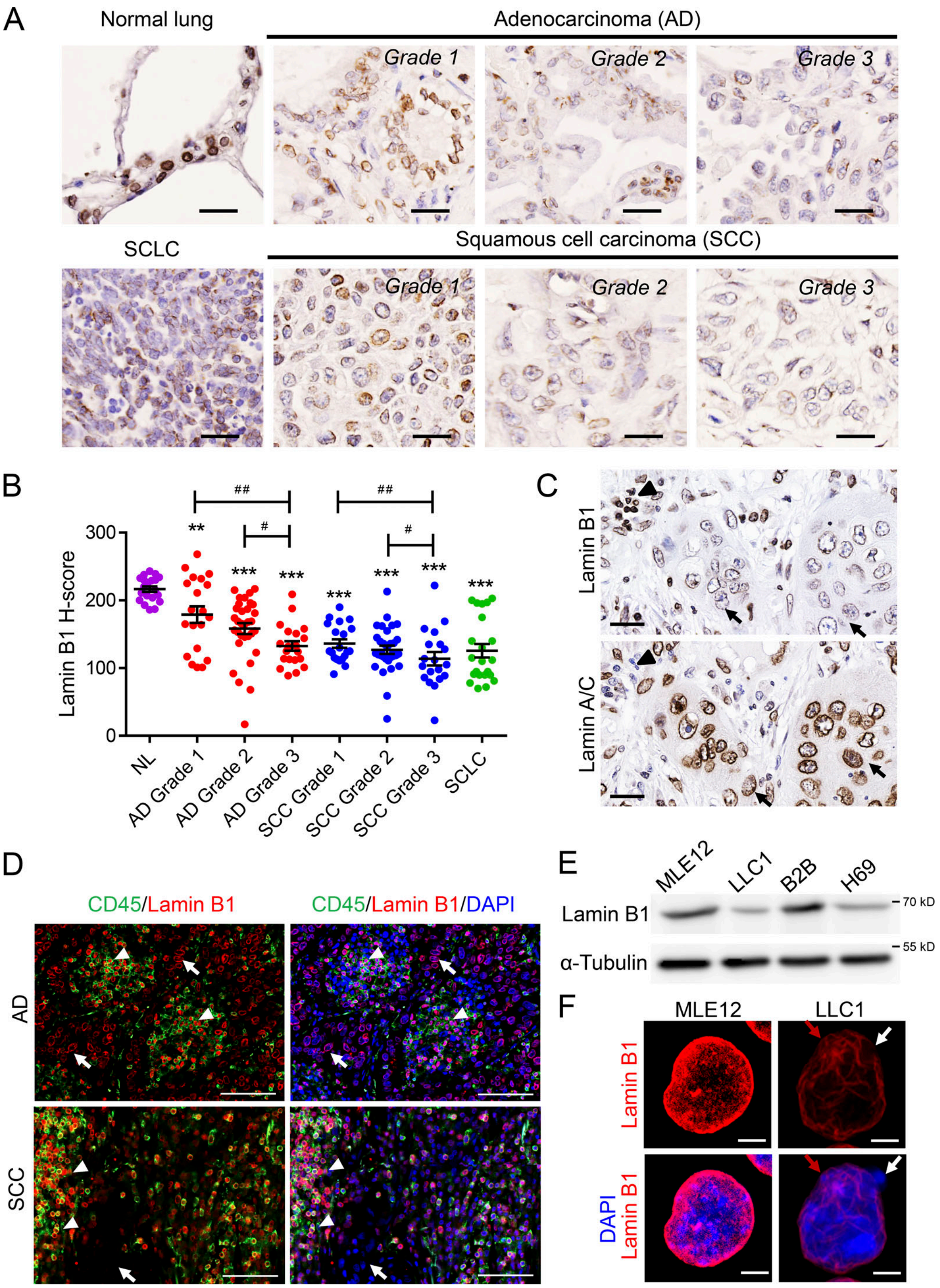

Figure S1. **Lamin B1 levels are decreased in lung cancers cells. (A)** IHC of representative tissue samples from different types and grades of lung tumors from a tissue microarray stained with a second anti-lamin B1 antibody (Santa Cruz; sc-6216), showing similar results to the IHC with the anti–lamin B1 antibody (Sigma-Aldrich; HPA050524) presented in Fig. 1. Scale bars, 25 µm. **(B)** Relative lamin B1 staining intensity (H-score) using an anti–lamin B1 antibody (Santa Cruz; sc-6216) in lung tumors of different types and grades. n = 70 for adenocarcinoma (AD; 20 grade 1, 30 grade 2, 20 grade 3); n = 69 for SCC (16 grade 1, 33 grade 2, 20 grade 3); n = 22 for SCLC; and n = 20 for normal lung tissue (NL). Statistical analysis was performed using Student's t test with two-tailed distribution. **, $P < 0.01$; ***, $P < 0.001$ versus normal lung tissue. #, $P < 0.05$; ##, $P < 0.01$. **(C)** IHC of consecutive lung cancer tissue samples using anti–lamin B1 antibody (Sigma-Aldrich; HPA050524) and lamin A antibody, showing high levels of lamin A and low levels of lamin B1 in lung cancer cells (arrows). Stroma cells (arrowheads) show high levels of lamin B1 and no lamin A. Scale bars, 25 µm. **(D)** Coimmunostaining with CD45 and lamin B1 antibodies of representative adenocarcinoma and SCC samples showing that cells highly positive for lamin B1 are CD45 positive immune/inflammatory cells (arrowheads); arrows indicate CD45-negative cells. Scale bars, 50 µm. **(E)** Immunoblot analysis of total cell lysates of mouse lung epithelial (MLE12) cells, mouse Lewis lung carcinoma (LLC1), human bronchial epithelium B2B, and H69 human SCLC cells showing decreased lamin B1 levels in mouse and human lung cancer cell lines. **(F)** Confocal images of immunostaining for lamin B1 in MLE12 and LLC1 cells. Scale bars, 5 µm. White arrows show nuclear blebs and local lamin B1 loss in LLC1 cells, whereas red arrows point to abnormalities in lamin B1 staining. To more clearly visualize the staining pattern in the LLC1 cells, which had a weaker lamin B1 signal, we acquired the LLC1 images with higher exposure compared with the MLE12 cells.

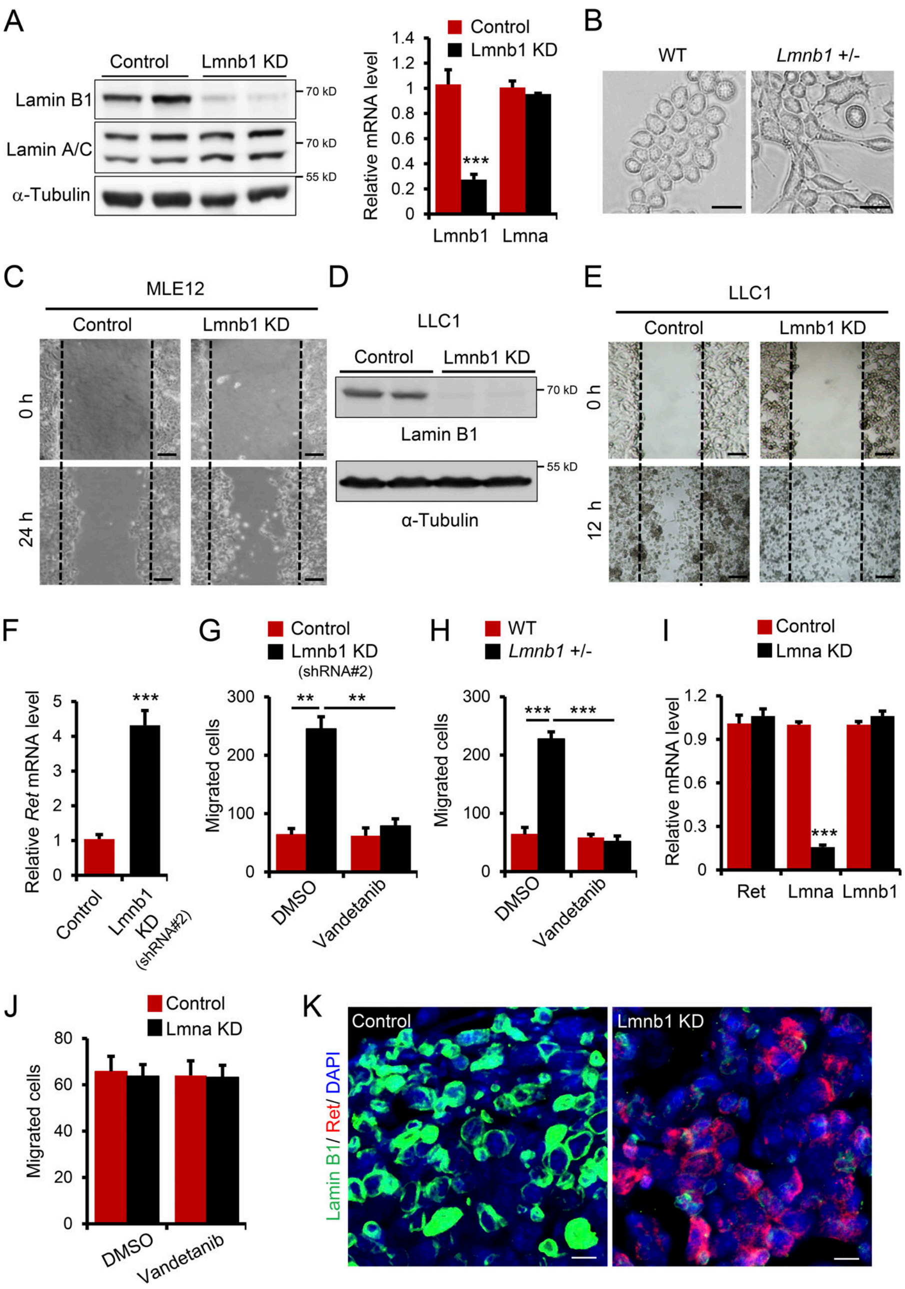

Figure S2. **Lamin B1 KD promotes cell migration and metastasis. (A)** Immunoblot analysis of total cell lysates of MLE12 cells expressing control shRNA or shRNA against lamin B1 using lamin B1 and lamin A/C antibodies. α-Tubulin served as loading control (left panel). qPCR analysis of *Lmnb1* and *Lmna* expression in MLE12 expressing control shRNA or shRNA against lamin B1 (right panel; *n* = 6). **(B)** Morphological changes in *Lmnb1*$^{+/-}$ MLE12 cells generated by CRISPR/Cas9 gene editing. Scale bars, 25 µm. **(C)** Scratch wound healing assay with control and lamin B1 KD MLE12 cells. Scale bars, 100 µm. **(D)** Immunoblot analysis of total cell lysates of LLC1 cells expressing control shRNA or shRNA against lamin B1. **(E)** Scratch wound healing assay with control and lamin B1 KD LLC1 cells. Scale bars, 100 µm. **(F)** qPCR validation of *Ret* upregulation upon lamin B1 depletion using a different shRNA against *Lmnb1* compared with the one from Fig. 3 D (*n* = 6). **(G and H)** Boyden chamber migration assay with control and lamin B1 KD MLE12 cells (using a different shRNA against *Lmnb1* compared with the one presented in Fig. 4 C; G) or with control and *Lmnb1*$^{+/-}$ MLE12 cells (H) treated either with DMSO or with 20 nM vandetanib (*n* = 4), validating our findings. **(I)** qPCR analysis of *Ret*, *Lmnb1*, and *Lmna* expression in MLE12 expressing control shRNA or shRNA against lamin A/C (*n* = 6). **(J)** Boyden chamber migration assay with control and lamin A/C KD MLE12 cells, treated either with DMSO or with 20 nM vandetanib (*n* = 4). **(K)** Co-staining with RET and lamin B1 antibodies of metastatic nodules in the lungs of mice injected with control or lamin B1 KD LLC1 cells. Scale bars, 10 µm. Data shown in A and F–J are mean ± SEM of a minimum of two independent experiments. Statistical analysis was performed using Student's *t* test with two-tailed distribution. **, P < 0.01; ***, P < 0.001.

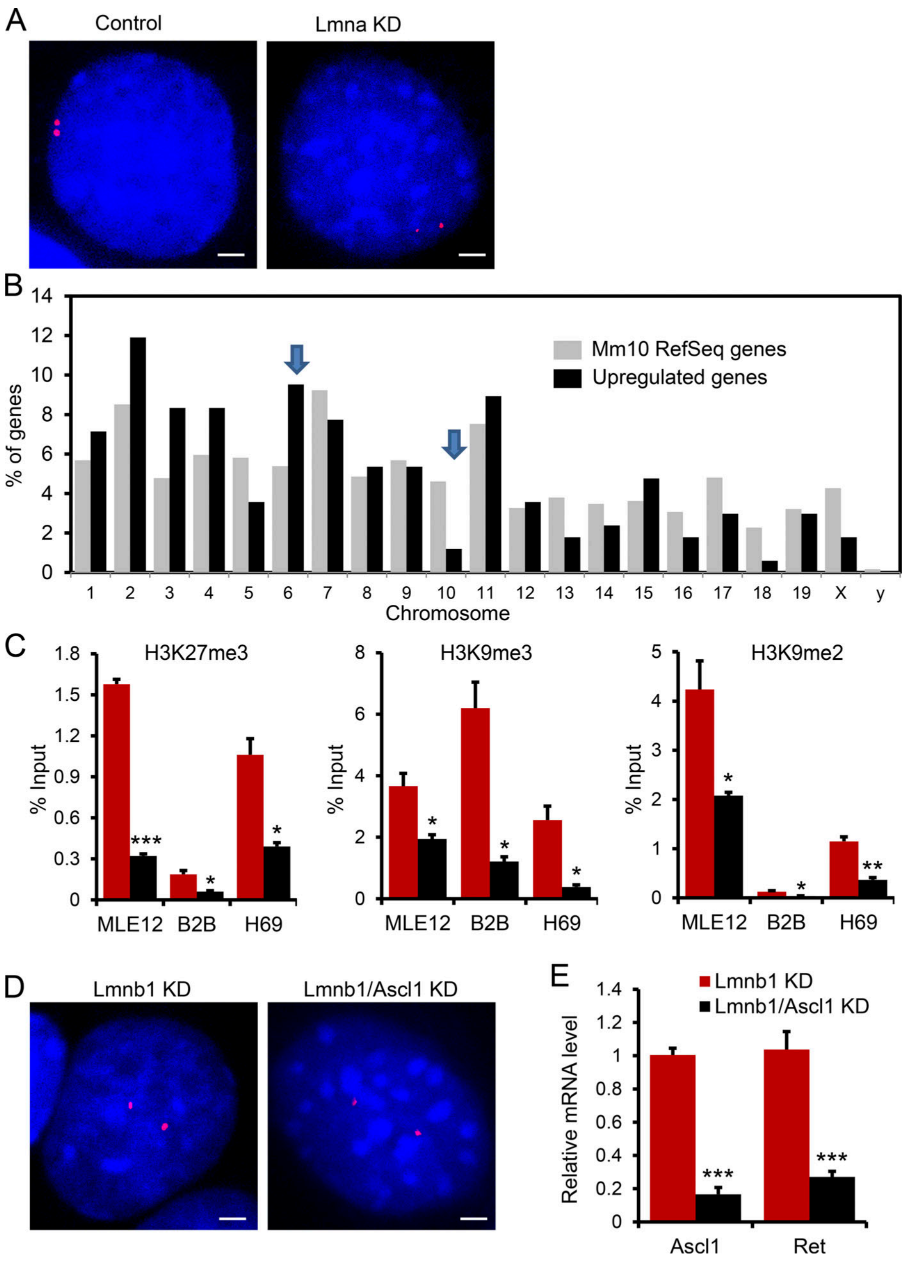

Figure S3. **Lamin B1 controls gene positioning and chromatin architecture in lung epithelial cells. (A)** Representative DNA FISH images of the *Ret* gene (red) in control or Lmna KD MLE12 cells. Cell nuclei were labeled with DAPI. Scale bars, 2 µm. **(B)** Distribution among chromosomes of differentially regulated genes upon lamin B1 loss of function (*n* = 2). Chr. 6 and 10 are indicated with arrows. **(C)** ChIP-qPCR analysis of H3K27me3, H3K9me3, and H3K9me2 occupancy at the *Gfra1* promoter (*n* = 4). **(D)** Representative DNA FISH images of the *Ret* gene (red) in Lmnb1 KD or Lmnb1/Ascl1 double-KD MLE12 cells. Scale bars, 2 µm. **(E)** qPCR analysis of *Ascl1* and *Ret* expression in Lmnb1 KD or Lmnb1/ Ascl1 double-KD MLE12 cells (*n* = 6). Images in A and D are representative of two independent experiments. Data shown in C and E are mean ± SEM of a minimum of two independent experiments. Statistical analysis was performed using Student's *t* test with two-tailed distribution. *, P < 0.05; **, P < 0.01; ***, P < 0.001.

https://doi.org/10.1084/jem.20181394

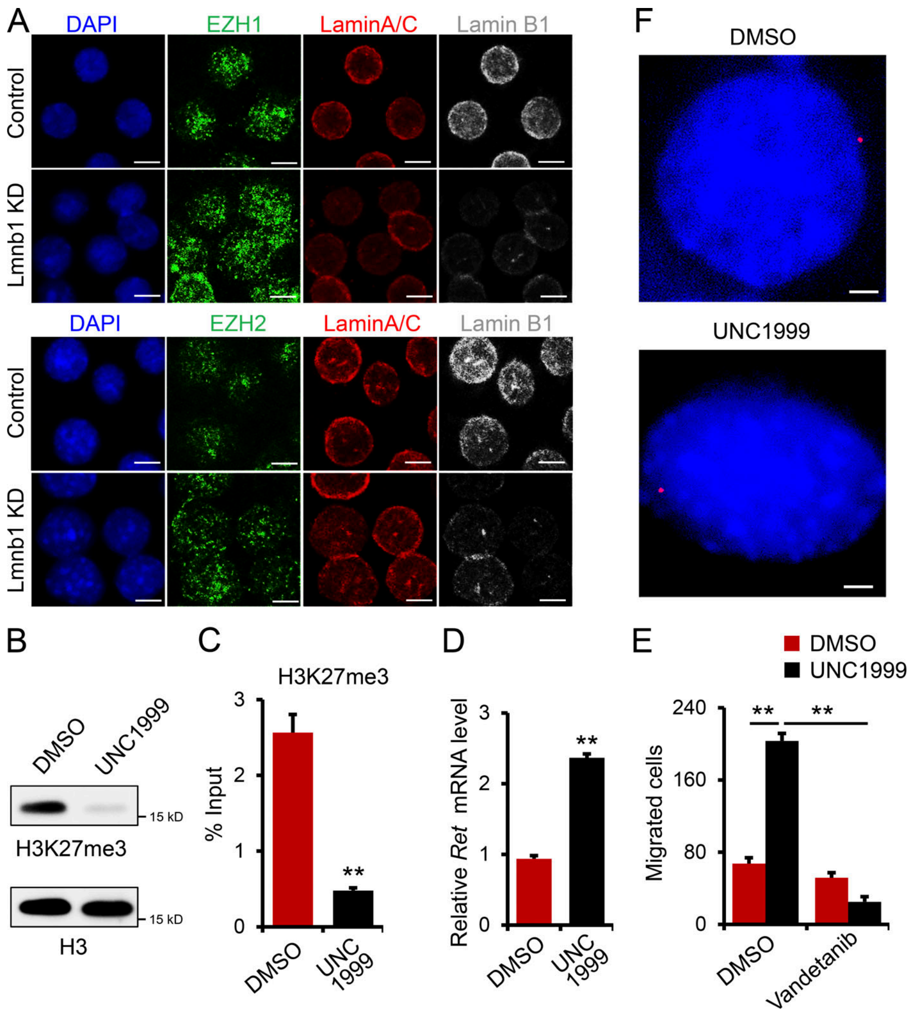

Figure S4. **Loss of PRC2 recruitment to chromatin induces a highly migratory phenotype in lung epithelial cells with decreased lamin B1 levels. (A)** Co-staining with lamin B1, lamin A/C, and EZH1 or EZH2 antibodies of cells treated with cytoskeletal buffer before in situ nuclear matrix extraction presented in Fig. 8 B. DNA content was assessed by DAPI staining. Scale bars, 5 µm. **(B)** Western blot analysis of global H3 and H3K27me3 levels in acid extracts of MLE12 cells treated with DMSO or the EZH1/EZH2 inhibitor UNC1999 (1 µM) for 3 d. **(C)** ChIP-qPCR analysis of H3K27me3 at the *Ret* promoter in MLE12 cells treated with DMSO or 1 µM UNC1999 (n = 4). **(D)** Relative *Ret* expression levels in MLE12 cells treated with DMSO or 1 µM UNC1999 (n = 6). **(E)** Boyden chamber migration assay with MLE12 cells treated with DMSO, 1 µM UNC1999, or 1 µM UNC1999 and 20 nM vandetanib (n = 4). **(F)** Representative DNA FISH images of the *Ret* gene (red) in MLE12 cells treated with DMSO or 1 µM UNC1999. Scale bars, 2 µm. Results (A–E) are representative of a minimum of two independent experiments. Images in F are representative of two independent experiments. Statistical analysis was performed using Student's t test with two-tailed distribution. Data are mean ± SEM. **, P < 0.01.

https://doi.org/10.1084/jem.20181394

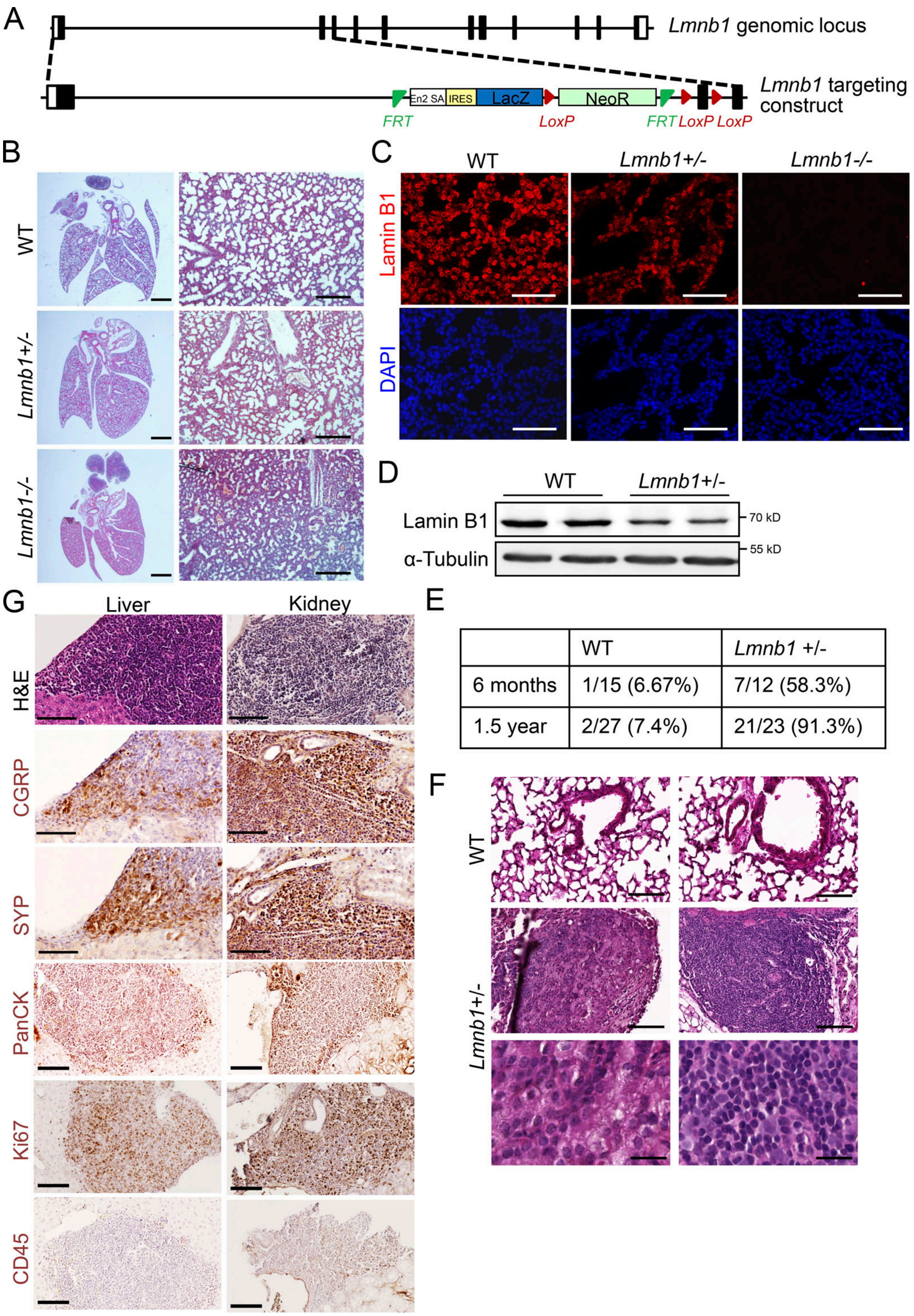

Figure S5. **Lung defects in *Lmnb1*$^{+/-}$ and *Lmnb1*$^{-/-}$ mice.** **(A)** Schematic representation of the *Lmnb1* genomic locus and *Lmnb1* targeting construct. **(B)** H&E staining of WT, *Lmnb1*$^{+/-}$, and *Lmnb1*$^{-/-}$ E18.5 lungs (n = 3). The right panels represent higher magnification images. Scale bars of left panels, 2 mm; scale bars of right panels, 100 µm. **(C)** Immunostaining of WT, *Lmnb1*$^{+/-}$, and *Lmnb1*$^{-/-}$ lungs with lamin B1 antibody, showing reduced lamin B1 expression in the heterozygous lungs and lack of lamin B1 expression in the knockout lungs (n = 3). Scale bars, 50 µm. **(D)** Western blot analysis of lysates from mouse embryonic fibroblasts derived from WT and *Lmnb1*$^{+/-}$ mice. **(E)** Spontaneous tumor incidence in lungs of WT and *Lmnb1*$^{+/-}$ littermates at 6 mo and 1.5 yr of age. **(F)** Histological analysis of representative lung sections of WT and *Lmnb1*$^{+/-}$ mice at 6 mo of age. Scale bars, 100 µm. **(G)** Histological and immunohistochemical analysis of liver and kidney of *Lmnb1*$^{+/-}$ mice at 1.5 yr of age. Scale bars, 100 µm.

Table S1. **Antibodies, chemicals, and plasmids used in this study**

Name	Source	Identifier
Lamin B (C-20) antibody	Santa Cruz Biotechnology	Cat# sc-6216; RRID: AB_648156
		Dilutions: WB 1:1,000; IF/IHC 1:100
LMNB1 antibody	Sigma-Aldrich	Cat# HPA050524; RRID: AB_2681156
		IF/IHC 1:100
Lamin A/C (N-18) antibody	Santa Cruz Biotechnology	Cat# sc-6215; RRID: AB_648152
		Dilutions: WB 1:1,000; IF/IHC 1:100
Polyclonal rabbit anti-cytokeratin	Dako	Cat# Z0622; RRID: AB_2650434
		Dilutions: IF/IHC 1:300
CD45 antibody	Abcam	Cat# ab10558; RRID: AB_442810
		Dilutions: IF/IHC 1:200
Anti-CGRP antibody produced in rabbit	Sigma-Aldrich	Cat# C8198; RRID: AB_259091
		Dilutions: IHC 1:100
Synaptophysin polyclonal antibody	Thermo Fisher Scientific	Cat# PA5-27286; RRID: AB_2544762
		Dilutions: IHC 1:100
Anti-CD3 mouse mAb (UCHT1) antibody	Millipore	Cat# 217570-100UG; RRID: AB_211569
		Dilutions: IF/IHC 1:200
Ki67 antibody	Abcam	Cat# ab15580; RRID: AB_443209
		Dilutions: IHC 1:200
PCNA (PC10) antibody	Santa Cruz Biotechnology	Cat# sc-56; RRID: AB_628110
		Dilutions: IHC 1:100
Napsin A antibody	Abcam	Cat# ab73021; RRID: AB_1269521
		Dilutions: IF 1:100
E cadherin antibody (DECMA-1)	Abcam	Cat# ab11512; RRID: AB_298118
		Dilutions: WB 1:2,000; IF 1:150
Fibronectin antibody	Abcam	Cat# ab2413; RRID: AB_2262874
		Dilutions: WB 1:1,000, IF 1:100
Mouse anti-N-cadherin mAb	Sigma-Aldrich	Cat# C3865; RRID: AB_262097
		Dilutions: WB 1:500; IF 1:100
Vimentin (D21H3) XP rabbit mAb antibody	Cell Signaling Technology	Cat# 5741; RRID: AB_10695459
		Dilutions: IF 1:100
Monoclonal anti-α-tubulin antibody produced in mouse	Sigma-Aldrich	Cat# T5168; RRID: AB_477579
		Dilutions: WB 1:8,000
Anti-Ret antibody (EPR2871)	Abcam	Cat# ab134100
		Dilutions: WB 1:2,000
Ret (C-19) antibody	Santa Cruz Biotechnology	Cat# sc-167; RRID: AB_631317
		Dilutions: IF/IHC 1:50
Phospho-p38 MAPK (Thr180/Tyr182; D3F9) XP rabbit mAb	Cell Signaling Technology	Cat# 4511; RRID: AB_2139682
		Dilutions: WB 1:1,000; IHC 1:100
Anti-p38 MAPK Antibody	Cell Signaling Technology	Cat# 9212; RRID: AB_330713
		Dilutions: WB 1:1,000
Phospho-SAPK/JNK (Thr183/Tyr185; G9) mouse mAb #9255 antibody	Cell Signaling Technology	Cat# 9255; RRID: AB_2307321
		Dilutions: WB 1:2,000
p-ERK (E-4) antibody	Santa Cruz Biotechnology	Cat# sc-7383; RRID: AB_627545
		Dilutions: WB 1:1,000

Table S1. **Antibodies, chemicals, and plasmids used in this study (*Continued*)**

Name	Source	Identifier
p-Ret (Tyr 1062)-R antibody	Santa Cruz Biotechnology	Cat# sc-20252-R; RRID: AB_2179766
		Dilutions: WB 1:1,000; IF/IHC 1:50
Rabbit anti-EZH1 polyclonal antibody	Abcam	Cat# ab13665; RRID: AB_300546
		Dilutions: WB 1:500; IF 1:100
KMT6/EZH2 antibody	Abcam	Cat# ab3748; RRID: AB_304045
		Dilutions: WB 1:1,000; IF 1:100
Histone H3 (dimethyl K9) antibody	Abcam	Cat# ab1220; RRID: AB_449854
Rabbit anti-histone H3, trimethyl (Lys9) ChIP grade polyclonal antibody	Abcam	Cat# ab8898; RRID: AB_306848
		Dilutions: PLA 1:100
H3K27me3–human, H3K27me3–mouse antibody	Millipore	Cat# 07-449; RRID: AB_310624
		Dilutions: WB 1:2,000; PLA 1:100
Histone H3 antibody–ChIP grade	Abcam	Cat# ab1791; RRID: AB_302613
		Dilutions: WB 1:8,000
Ascl1 antibody	BD Biosciences	Cat# 556604; RRID: AB_396479
RNA polymerase II CTD repeat YSPTSPS antibody (8WG16)	Abcam	Cat# ab817; RRID: AB_306327
Sheep anti-digoxigenin Fab fragments antibody, rhodamine conjugated	Roche	Cat# 11207750910; RRID: AB_514501
Vandetanib (ZD6474)	Selleckchem	Cat# S1046
SB202190	Adipogen	Cat# AG-CR1-0028
UNC1999	Sigma-Aldrich	Cat# 1431612-23-5
Matrigel GFR basement membrane matrix	Corning	Cat# 354230
pLKO.1—mouse Ret shRNAs	Sigma-Aldrich	SHCLNG-NM_009050
		Clone ID: NM_009050.1-2934s1c1
pLKO.1—mouse Lmnb1 shRNAs #1	Sigma-Aldrich	SHCLNG-NM_010721
		Clone ID: NM_010721.1-675s1c1
pLKO.1—mouse Lmnb1 shRNAs #2	Sigma-Aldrich	SHCLNG-NM_010721
		Clone ID: NM_010721.1-675s1c1
pLKO.1—human Lmnb1 shRNAs	Sigma-Aldrich	SHCLND-NM_005573
		Clone ID: NM_005573.2-760s1c1
pLKO.1—mouse Lmna shRNAs	Sigma-Aldrich	SHCLNG-NM_001002011.2
		Clone ID: NM_001002011.1-1627s1c1
pLKO.1—mouse Ascl1 shRNAs	Sigma-Aldrich	SHCLNG-NM_008553.4
		Clone ID: NM_008553.2-670s1c1
pLKO.1—mouse Ezh1 shRNAs	Sigma-Aldrich	SHCLND-NM_007970
		Clone ID: NM_007970.1-1805s1c1
pLKO.1—mouse Ezh2 shRNAs	Sigma-Aldrich	SHCLND-NM_007971
		Clone ID: NM_007971.1-1497s1c1

Cat#, catalog number; GFR, growth factor reduced; IF, immunofluorescence; WB, Western blot.
